# Generation of functionally competent testicular somatic cells from pluripotent stem cells

**DOI:** 10.1126/sciadv.adz0269

**Published:** 2026-02-26

**Authors:** Takuya Sato, Takashi Yoshino, Mai Ohtsuka, Takahiro Suzuki, Takafumi Matsumura, Yuki Matsudaira, Yu Ishikawa-Yamauchi, Shiori Maeda, Haruka Yabukami, Yoshiakira Kanai, Miki Inoue, Yuichi Shima, Makoto Tachibana, Shogo Matoba, Kimiko Inoue, Narumi Ogonuki, Atsuo Ogura, Katsuhiko Hayashi, Takehiko Ogawa

**Affiliations:** ^1^ Department of Regenerative Medicine, Yokohama City University Graduate School of Medicine, Yokohama 236-0004, Kanagawa, Japan.; ^2^ Department of Genome Biology, Graduate School of Medicine, Osaka University, Suita 565-0871, Osaka, Japan.; ^3^ Premium Research Institute for Human Metaverse Medicine (WPI-PRIMe), Osaka University, Suita 565-0871, Osaka, Japan.; ^4^ Laboratory for Cellular Function Conversion Technology, RIKEN Center for Integrative Medical Sciences, Yokohama 230-0045, Kanagawa, Japan.; ^5^ Functional Genomics, Graduate School of Medical Life Science, Yokohama City University, Yokohama 230-0045, Kanagawa, Japan.; ^6^ Department of Obstetrics & Gynecology, Juntendo University Faculty of Medicine, Tokyo, Japan.; ^7^ Department of Veterinary Anatomy, The University of Tokyo, Bunkyo-ku, Tokyo 113-8657, Japan.; ^8^ Department of Anatomy, Kurume University School of Medicine, 67 Asahi-machi, Kurume 830-0011, Fukuoka, Japan.; ^9^ Laboratory of Epigenome Dynamics, Graduate School of Frontier Biosciences, Osaka University, Suita 565-0871, Osaka, Japan.; ^10^ Bioresource Engineering Division, BioResource Research Center, RIKEN, Tsukuba 305-0074, Ibaraki, Japan.; ^11^ Germline Genetics, Graduate School of Frontier Biosciences, Osaka University, Suita 565-0871, Osaka, Japan.

## Abstract

Cellular interactions between germ cells and gonadal somatic cells are essential for the progression of gametogenesis. Here, we report a culture method for generating fetal testicular somatic cell–like cells (fTeSLCs) from embryonic stem cells. These fTeSLCs exhibit a transcriptomic profile closely resembling that of their in vivo counterparts, including distinct cell populations corresponding to Sertoli cells and interstitial cells. For functional assessment, interstitial cell–like cells (ICLCs) and Sertoli-like cells (SerLCs) were isolated from fTeSLCs. ICLCs differentiated into Leydig cells when cocultured with testes lacking endogenous Leydig cells, thereby restoring androgenic support. SerLCs reconstituted the seminiferous epithelium following selective ablation of endogenous Sertoli cells. Both cell types supported spermatogenesis and generated spermatids reaching the elongating stage. Notably, round spermatids derived from these reconstructed systems produced viable offspring by round spermatid injection. These findings demonstrate that fTeSLCs can generate functional testicular somatic cells, providing a valuable platform for studying testis development and spermatogenesis.

## Teaser

Stem cells give rise to testicular cells that bring the sperm-making machinery back online.

## INTRODUCTION

The testis is a vital organ primarily composed of seminiferous tubules, where spermatogenesis takes place. Within these tubules, Sertoli cells are the only somatic cells present. They form tight junctions with one another, establishing the blood-testis barrier. Each spermatogenic cell maintains direct contact with a Sertoli cell, a crucial interaction necessary for the proper progression of spermatogenesis (*1*). Beyond Sertoli cells, the testicular environment hosts various other somatic cell types that support spermatogenesis. These include peritubular myoid cells, which surround the seminiferous tubules, as well as Leydig cells, which produce testosterone essential for spermatogenesis and male secondary sexual characteristics, and macrophages, which reside in the interstitial space and contribute to testicular function (*2*).

The gonad originates from the genital ridge, which emerges as a thickening of the coelomic epithelium on the ventromedial surfaces of the mesonephroi around embryonic day 9 (E9). The expression of Sry, the sex-determining gene located on the Y chromosome, in cells destined to become Sertoli cells (pre-Sertoli cells) directs the differentiation of the indifferent gonad into the testis around E11 (*3*–*5*). Consequently, Sertoli cells play a critical role not only in spermatogenesis but also in sex differentiation, while Leydig cells rapidly emerge in parallel and contribute indispensably to masculinization through hormone production.

Recent studies have reported culture methods for inducing the differentiation of mouse embryonic stem cells (ESCs) into testicular somatic cells (*6*–*11*). In addition, one study demonstrated the successful induction of Sertoli cells from mouse fibroblasts through direct reprogramming (*12*). These studies suggested that testicular somatic cells, particularly Sertoli cells, derived via in vitro induction exhibit gene expression profiles and other characteristics similar to their in vivo counterparts. However, none has yet determined whether these cells have genuine functional capabilities. In particular, their ability to support spermatogenesis, a fundamental function of Sertoli cells, has not been thoroughly investigated or demonstrated, and the functional potential of Leydig cells generated in vitro remains almost unexplored.

A major breakthrough in this field came with the establishment of a method for inducing the differentiation of ESCs into fetal ovarian somatic cell–like cells (FOSLCs) (*13*). These FOSLCs were cocultured with primordial germ cell–like cells, also derived from ESCs, to reconstruct ovarian tissue in vitro. Within this recreated tissue, oogenesis took place, leading to the successful generation of functional oocytes capable of fertilization, achieved entirely through in vitro processes. These findings demonstrate that FOSLCs function as ovarian somatic cells with sufficient capacity to support complete oogenesis. In the present study, we aimed to develop a method for differentiating male ESCs into functional embryonic testicular somatic cells, building upon the approach used for FOSLCs.

## RESULTS

### Establishment of reporter ESCs for testicular somatic cells

To monitor the differentiation of ESCs into testicular somatic cells, we established an ESC line carrying two reporter genes, *Nr5a1* and *Sry-box 9* (*Sox9*). *Nr5a1* is expressed in all cell lineages of the genital ridge (*14*, *15*), whereas *Sox9* is expressed exclusively in Sertoli cells in the gonad (*16*). We used a male XY ESC line established from *Nr5a1*-hCD271 transgenic (Tg) mice (*13*, *17*), into which a gene cassette consisting of the Clover green fluorescent protein (CGFP) gene, linked to a 2A peptide sequence at the 3′ end of the Sox9 gene, was inserted. This resulted in the establishment of *Nr5a1*-hCD271/*Sox9*-CGFP (N271S9C) ESCs (fig. S1A). To validate the expression patterns of these reporter genes in the embryonic testes, N271S9C ESCs were injected into wild-type (WT) eight-cell embryos, which were cultured to the blastocyst stage and subsequently transplanted into the uterus of host mice to produce chimeric embryos. In E12.5 chimeric embryos, CGFP expression was observed throughout the entire embryonic body, mirroring the pattern seen in *Sox9*-EGFP knockin mice (*18*). While this reflects the known expression of *Sox9* in certain embryonic tissues, including the central nervous system and chondrogenic regions, the strong fluorescence signal in the testis, particularly in regions corresponding to the seminiferous tubules, highlights its primary role in marking Sertoli cells (fig. S1B). To further characterize testicular cell populations, testes from chimeric embryos were collected and analyzed via flow cytometry (FCM) (fig. S1C). The *Sox9*-CGFP^high^ and *Nr5a1*-hCD271^high^ cell population was identified as Sertoli cells on the basis of the expression of these two markers and the absence of platelet-derived growth factor receptor–α (PDGFRα), a marker for testicular interstitial cells (*19*, *20*). The *Nr5a1*-hCD271^low^ cell population was classified as interstitial cells, including Leydig progenitors, as they were *Sox9*-CGFP–negative and PDGFRα-positive. To confirm these findings, immunostaining was performed on E12.5 testes obtained from the offspring of N271S9C chimeric mice. GFP-positive cells were confirmed as Sertoli cells on the basis of the coexpression of hCD271 and endogenous Sox9 (fig. S1D). In addition, PDGFRα expression was detected in GFP-negative interstitial cells, consistent with the FCM results (fig. S1E). These findings demonstrate the utility of N271S9C ESCs as a reporter system for testicular somatic cells, particularly for Sertoli cells.

### Induction of testicular somatic cells from XY ESCs using the method for FOSLC induction

**Fig. 1.**
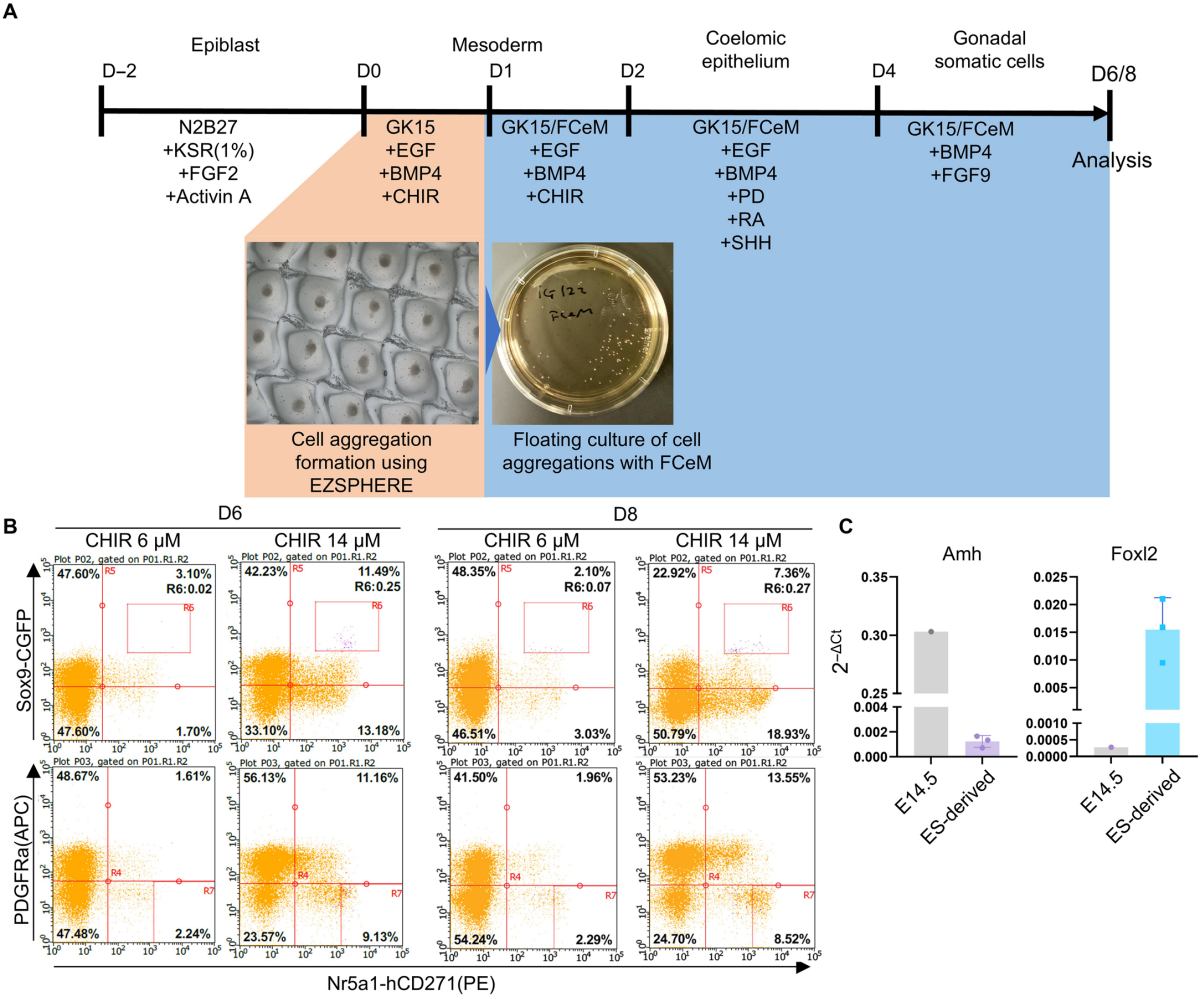
Induction of testicular somatic cells from XY ESCs using the method for FOSLC induction. (**A**) Schematic diagram showing the workflow of the method for the induction of GSCs from ESCs. (**B**) FCM analysis of ESC-derived induced cells. On D6 and D8, the cultured cells were stained with anti–hCD271-PE and anti–PDGFRα-APC antibodies and analyzed by FCM. A sufficient concentration of CHIR, a Wnt activator, was critical for maintaining the *Nr5a1*-hCD271^high^ fraction and *Nr5a1*-hCD271^low^/PDGFRa^+^ fraction. (**C**) *Nr5a1*-hCD271^+^ cells were sorted on culture D8, and qPCR was performed for *Amh* and *Foxl2*. Relative expression is measured as 2^−ΔCt^ values, normalized against β-actin. The expression levels of each gene were compared with those in E14.5 fetal tests. Values are the means ± SD of three independent experiments.

The induction of ovarian somatic cells from ESCs involves an initial step of generating epiblast-like cells (EpiLCs) from XX ESCs (*21*), followed by three sequential steps of medium replacement to promote differentiation into E12.5-equivalent ovarian somatic cells (*13*). The differentiation of EpiLCs into the nascent mesoderm relies on both a low concentration of bone morphogenetic protein 4 (BMP4) and the potent activation of Wnt signaling by CHIR99021 (CHIR). In the subsequent step, differentiation into the intermediate mesoderm/lateral plate mesoderm, which contains progenitors of the genital ridge, is induced by retinoic acid, PD0325901 (PD), and Sonic hedgehog. Last, it has been shown that differentiation into ovarian somatic cells can be induced with BMP4 and fibroblast growth factor 9 (FGF9). In the present study, we applied this method to XY N271S9C ESCs with the aim of inducing testicular somatic cell differentiation. It should be noted here that we made modifications to the original method to make it less laborious and applicable for a larger-scale experiment by introducing an EZSPHERE culture dish and floating culture method as described in detail in Materials and Methods (Fig. 1A). Using this approach, we successfully induced a large number of *Nr5a1*-hCD271^+^ cells by day 6 (D6), consistent with the previous report. Notably, among the supplemented factors, CHIR played the most critical role in inducing *Nr5a1*-hCD271 expression (Fig. 1B). However, *Sox9*-CGFP expression remained insufficient on both D6 and D8 (Fig. 1B), indicating that while gonadal somatic cell (GSC) differentiation occurred, further maturation into Sertoli cells was incomplete. To investigate the underlying cause, we isolated *Nr5a1*-hCD271^+^ cells from D8 cellular aggregates via fluorescence-activated cell sorting (FACS) and analyzed the expressions of *Anti-Mullerian hormone* (*Amh*), a marker of Sertoli cells, and *Forkhead box L2* (*Foxl2*), a marker of ovarian granulosa cells, by reverse transcription quantitative polymerase chain reaction (RT-qPCR). Cell lysates prepared from E14.5 embryonic testes were used in RT-qPCR for comparison. The result revealed that *Amh* expression was not fully induced, whereas *Foxl2* was overexpressed (Fig. 1C), suggesting that despite their XY genotype, the ESC-derived cells exhibited a GSC fate with a female-like character.

### Suppression of Wnt signaling induces Sertoli cell specification

**Fig. 2.**
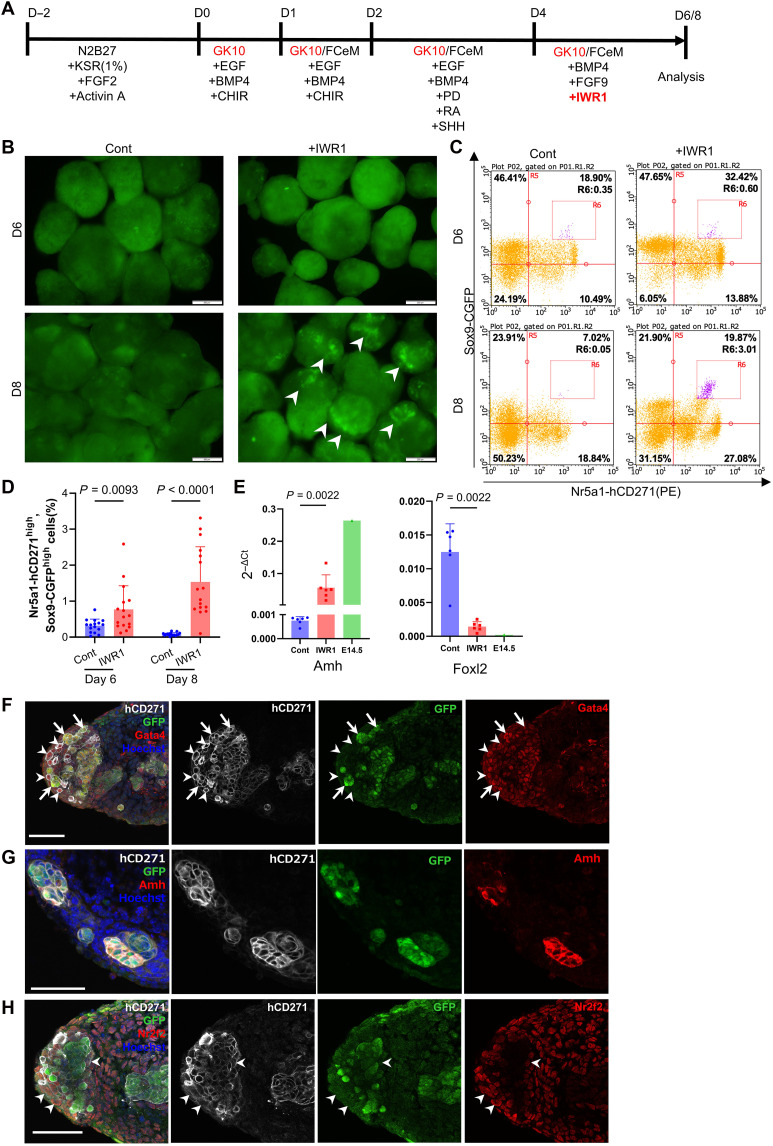
Induction of testicular somatic cell differentiation by suppression of Wnt signaling. (**A**) Schematic diagram showing the workflow of the final method for inducing gonadal cells from ES cells. IWR1, a Wnt inhibitor, was added to the D4 culture medium. (**B**) Cell aggregates observed by fluorescence microscopy. Weak CGFP expression was observed in the control (Cont) group. Cells with strong CGFP expression were detected in the cell aggregates of the IWR1-treated group (arrowheads) on D8. Scale bars, 200 μm. (**C**) FCM analysis of cells induced from ESCs with or without IWR1 treatment. Cells were labeled with an anti–hCD271-PE antibody on D6 and D8 and analyzed by FCM. (**D**) Induction efficiency of Sertoli cells, defined as *Nr5a1*-hCD271^high^/*Sox9*-CGFP^high^ cells, as assessed by FCM in control versus IWR1-treated groups. The bar graph shows the means ± SD of the percentage of Sertoli cells among all ESC-derived cells. Each dot represents an independent experiment. Statistical significance was assessed using the Mann-Whitney test. (**E**) *Nr5a1*-hCD271^+^ cells were sorted on culture D8, and RT-qPCRs for *Amh* and *Foxl2* were performed. Relative expression is measured as 2^−ΔCt^ values, normalized against β-*actin*. Values represent the means ± SD from six independent experiments. Statistical significance was assessed using the Mann-Whitney test. (**F** to **H**) Immunostaining of cryosections from cell aggregates cultured with IWR1 on D8. Aggregates were stained with antibodies against hCD271 and GFP, counterstained with Hoechst, and additionally stained with an antibody against Gata4 (F), Amh (G), or Nr2f2 (H). (F) Arrows indicate cells positive for Gata4, hCD271, and GFP; arrowheads indicate cells positive for Gata4 and hCD271 but negative for GFP. (H) Some hCD271-positive cells expressed Nr2f2 (arrowheads); these Nr2f2-positive cells were GFP-negative. Scale bars, 50 μm.

On the basis of the above findings, directing the sex differentiation of in vitro–formed gonad toward the male pathway appeared necessary. To achieve this, we focused on two key factors involved in gonadal sex differentiation. The first, Fgf9, plays a crucial role in testicular development. It is primarily known for maintaining the expression of the key male sex-determining genes, such as Sox9, and suppressing female sex-determining genes, including Wnt4, Rspo1 (R-spondin 1), and Foxl2, thereby stabilizing the male fate (*22*, *23*). The second, Wnt4, is essential for suppressing masculinization and directing XX gonads toward ovarian somatic cell differentiation, making Wnt signaling a recognized feminization signal (*22*, *24*, *25*). Thus, we first increased the FGF9 concentration in the medium at D4 from 2 to 100 ng/ml; however, no notable changes were observed (fig. S2). We then added IWR1 (Fig. 2A), a low-molecular compound inhibiting the Wnt signaling pathway, to the D4 medium. IWR1 functions by stabilizing the Axin complex, which targets β-catenin, a key intracellular mediator of the canonical Wnt signaling cascade (*26*). Upon IWR1 treatment, strong CGFP expression was observed in the D8 cell aggregates (Fig. 2B). An FCM analysis confirmed that in the absence of IWR1, almost no *Nr5a1*-hCD271^high^/*Sox9*-CGFP^high^ cells, indicative of Sertoli cells, were found. In contrast, IWR1 treatment led to an increase in this population at D6, which became more pronounced by D8 (Fig. 2, C and D). Furthermore, RT-qPCR analysis revealed that in the IWR1-treated group, the expression of fetal testis-specific genes (*Amh*, *Dhh*, and *Hsd17b3*) increased, while ovary-specific genes (*Foxl2*, *Fst*, and *Rspo1*) were suppressed (Fig. 2E and fig. S3). These results indicated that suppression of Wnt signaling by IWR1 effectively promoted masculinization of in vitro–formed gonads, leading to the emergence of Sertoli-like cells (SerLCs).

To examine the histological expression patterns of representative marker genes, we performed immunostaining on cryosections of the D8 cell aggregates. We confirmed that hCD271-positive (i.e., *Nr5a1*-hCD271–positive) cells included GFP-positive (i.e., *Sox9*-CGFP–positive) cells, which also expressed endogenous Nr5a1 and Sox9 (fig. S4). In addition, hCD271-positive cells expressed Gata4, a marker for both Sertoli and interstitial cells in the fetal testis (*27*). Within the hCD271-positive/Gata4-positive population, some cells were also GFP-positive, indicating their identity as putative Sertoli cells (Fig. 2F and fig. S4C). Furthermore, cells that were hCD271-positive/GFP-positive and expressed Amh, a Sertoli cell marker (*28*), were observed (Fig. 2G and fig. S4C).

Next, we assessed the expression of nuclear receptor subfamily 2 group F member 2 (Nr2f2; also known as Coup-TFII), a key regulator of Leydig cell differentiation and a marker for interstitial cells and their progenitors (*29*–*32*). Nr2f2 positivity was observed in hCD271-positive/GFP-negative cells in the cell aggregates but not in hCD271-positive/GFP-positive cells (putative Sertoli cells), as anticipated (Fig. 2H and fig. S4C). These results suggest that ESCs differentiated into various types of testicular somatic cells, including Sertoli and interstitial cells. In addition to applying IWR1 in the D4 medium, we ultimately found that the optimal concentration of KnockOut Serum Replacement (KSR) was 10%, whereas our experiments were initially conducted with 15% (appearing as GK10 in Fig. 2A).

To confirm that our protocol is robustly applicable to other ESC lines, we used a different line of *Nr5a1*-hCD271 and a line derived from *Amh*-DTR mouse, which has a different genetic background (ICR, C57BL/6, and their mixture) from the *Nr5a1*-hCD271 line. In both cases, ESC aggregates were applied with the induction protocol (Fig. 2A), and the D8 samples were analyzed with immunostaining. Samples from another *Nr5a1*-hCD271 cell line showed a regional positive area to hCD271, Nr5a1, Sox9, and Amh, indicating successful differentiation to supporting cells including putative Sertoli cells (fig. S5, A and B). Samples from the cell line of *Amh*-DTR also showed Nr5a1, Sox9, and Amh, indicating that the induction to testicular somatic cells was faithfully repeatable in other ECS lines (fig. S5, C and D).

### The in vitro–formed gonadal cells showed a transcriptomic profile similar to in vivo testicular somatic cells

**Fig. 3.**
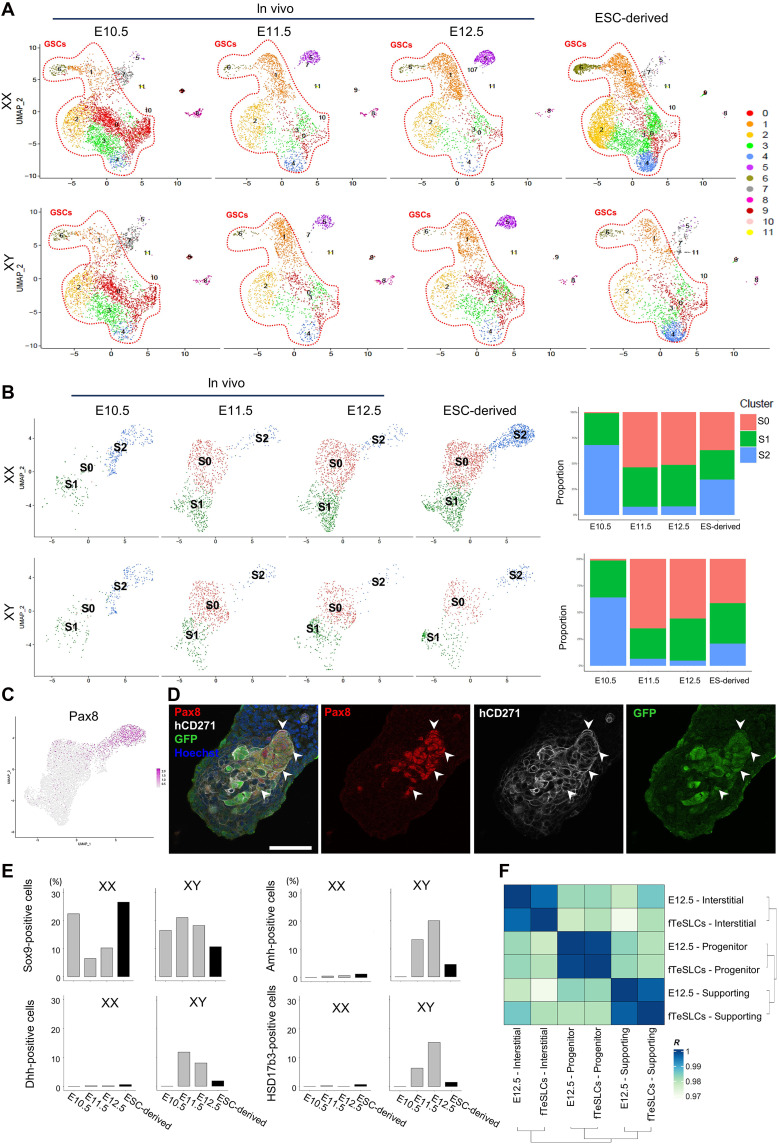
Single-cell analysis of in vivo gonadal cells and in vitro ESC-derived cells. (**A**) UMAP plot of in vivo gonadal cells and MACS-sorted *Nr5a1*-hCD271–positive cells at D6 from male and female ESC-derived cultures. Red dotted lines indicate clusters identified as putative GSCs. (**B**) UMAP plot of supporting cells. The clusters classified as supporting cells in (B) were reanalyzed and divided into three clusters. The graph on the right shows the percentage of each cluster in each sample. (**C**) UMAP plot visualizing *Pax8* expression within the supporting cell clusters, with expression levels shown as a magenta color gradient. (**D**) Fluorescence immunostaining of D8 cell aggregates cultured in IWR1-supplemented medium, showing the expression of hCD271, GFP, and Pax8, with Hoechst used for nuclear staining. Arrowheads indicate supporting-like lineage cells expressing Pax8, CD271, and GFP. Scale bar, 50 μm. (**E**) Bar graph showing the percentage of *Sox9*-, *Amh*-, *Dhh*-, and *Hsd17b3*-expressing cells within the supporting cell clusters identified in (C). (**F**) Heatmap of correlation analysis for testicular somatic cell clusters, comparing those from in vivo embryonic testis and ESC-derived gonadal cells (fTeSLCs), ordered on the basis of hierarchical clustering.

To characterize the testicular somatic cell–like cells induced from XY ESCs in vitro, *Nr5a1*-hCD271^+^ cells were enriched from about 14% initially to 40% using magnetic-activated cell sorting (MACS) at D6 (fig. S6, A to C), as described in our previous study (*13*). We then performed single-cell RNA sequencing (scRNA-seq). The transcriptomic profiles were compared with those of male and female gonadal cells from E10.5, E11.5, and E12.5 embryos, as well as with ESC-derived cells from XX ESCs (*13*). Cell clustering was conducted on all male and female gonadal cells obtained in vivo and ESC-derived samples, followed by dimensionality reduction using uniform manifold approximation and projection (UMAP) to visualize the data in the two-dimensional space (fig. S7A). The UMAP plot of cells induced from XY ESCs exhibited a pattern similar to that of FOSLCs derived from XX ESCs (Fig. 3A) and closely resembled in vivo E11.5 or E12.5 gonadal cells. Among the 12 clusters, several were annotated on the basis of specific marker gene expression (fig. S7B). The clusters other than 5 and 7 (primordial germ cells/germ cells), 8 (endothelial cells), 9 (erythroid cells), and 11 (megakaryocytes) were considered collectively as GSCs (Fig. 3A and fig. S7, A and B). The proportion of cells expressing endogenous *Nr5a1* was consistently around 30% across all examined GSC populations, including both in vivo and ESC-derived samples, with no notable variation observed (fig. S7C).

To analyze the transcriptome data in depth, clustering and UMAP dimensionality reduction were performed specifically for GSCs. This analysis reclassified the cells into seven clusters (G0 to G6) (fig. S8A). In the UMAP plot, some differences in distribution were recognizable between XX and XY samples, as well as between in vivo and in vitro samples, although these differences were not pronounced. Each group was broadly distributed across all cluster regions (fig. S8B). We therefore proceeded to characterize each cluster on the basis of the expression profile of key marker genes defined in previous transcriptome studies (*13*, *29*). Clusters G1 and G6 were identified as supporting cell clusters, encompassing both Sertoli and granulosa lineages (fig. S8C). Because these populations have not yet diverged clearly at this stage, they appear together in the UMAP representation. Clusters G4 and G5 strongly expressed interstitial cell genes such as *Pdgfr*α, *Tcf21*, *Arx26, Sanil2, Mafb*, and *Nr2f2* (fig. S8C), suggesting that they were interstitial cells. The remaining clusters G0, G2, and G3 exhibited gene expression patterns, suggesting progenitor cell–like characteristics, including *Upk3b, Tmem151a*, and *Tm4sf5* (fig. S8C) (*13*, *14*).

To further characterize the supporting cells, we performed additional reclustering and UMAP analyses, identifying three distinct subclusters: S0, S1, and S2 (Fig. 3B). In the in vivo dataset, cells classified into S1 and S2 were already present at E10.5, followed by the emergence of S0 at E11.5 (Fig. 3B). Clusters S0 and S1, especially S0 appearing at E11.5, constitute a population differentiating into granulosa cells, marked by *Fst* (fig. S9A), or Sertoli cells (fig. S10). On the other hand, S2 displayed unique characteristics, including its early appearance at E10.5, a subsequent reduction in relative proportion compared to S0 and S1, and distinct gene expression patterns marked by *Pax8*, *Id1*, and *Id2* (Fig. 3, B and C, and fig. S9A). These findings suggest that S2 cells belong to a recently identified lineage known as supporting-like lineage cells (*33*). Notably, ESC-derived S2 cluster cells exhibited a gene expression pattern similar to their in vivo counterparts at E11.5 to E12.5 (fig. S9B). Furthermore, immunofluorescence staining confirmed that D6 and D8 aggregates contained paired box 8 (Pax8)–expressing cells among hCD271^+^/GFP^+^ cells, corresponding to supporting-like cells (Fig. 3D and fig. S11).

Then, to assess whether differentiation into testicular somatic cells proceeded properly, we examined the expression of male-specific genes in supporting cell clusters using scRNA-seq data (Fig. 3E and fig. S10). Unexpectedly, Sox9 expression was detected not only in cells derived from XY ESCs but also in a larger proportion of cells derived from XX ESCs. This observation likely reflects the relatively high proportion of cluster S2 cells, corresponding to supporting-like cells, among those derived from XX ESCs (Fig. 3B). A previous study reported that supporting-like lineage cells in both male and female gonads express Sox9 (*33*), supporting this interpretation. In contrast, *Amh*, *Desert hedgehog* (*Dhh*), and *Hydroxysteroid 17-beta dehydrogenase 3* (*Hsd17*β*3*), male-specific genes expressed in Sertoli cells, were primarily detected in clusters S0 and S1 of XY E11.5 and E12.5 cells (fig. S10). However, cells expressing all four genes, or three of them concomitantly, were rare (fig. S12). Moreover, the proportions of these gene-expressing cells in ESC-derived XY samples were lower than those in their in vivo counterparts, although still higher than in ESC-derived XX samples (Fig. 3E). These findings indicate that at D6 in culture, induction of XY ESCs into SerLCs was still incomplete and ongoing.

Last, we compared the gene expression profiles of interstitial cells [clusters G4 and G5 in fig. S8 (A and B)], progenitor cells [clusters G0, G2, and G3 in fig. S8 (A and B)], and supporting cells [clusters G1 and G6 in fig. S8 (A and B)] between E12.5 in vivo XY gonadal cells and XY ESC-derived cells. We observed a strong correlation across all three populations (Fig. 3F). These findings indicate that the cells generated from the XY ESCs contained various cell types composing the embryonic testis. We refer to these cells as fetal testicular somatic cell–like cells (fTeSLCs).

### Interstitial cell–like cells in fTeSLCs can differentiate into functional Leydig cells

**Fig. 4.**
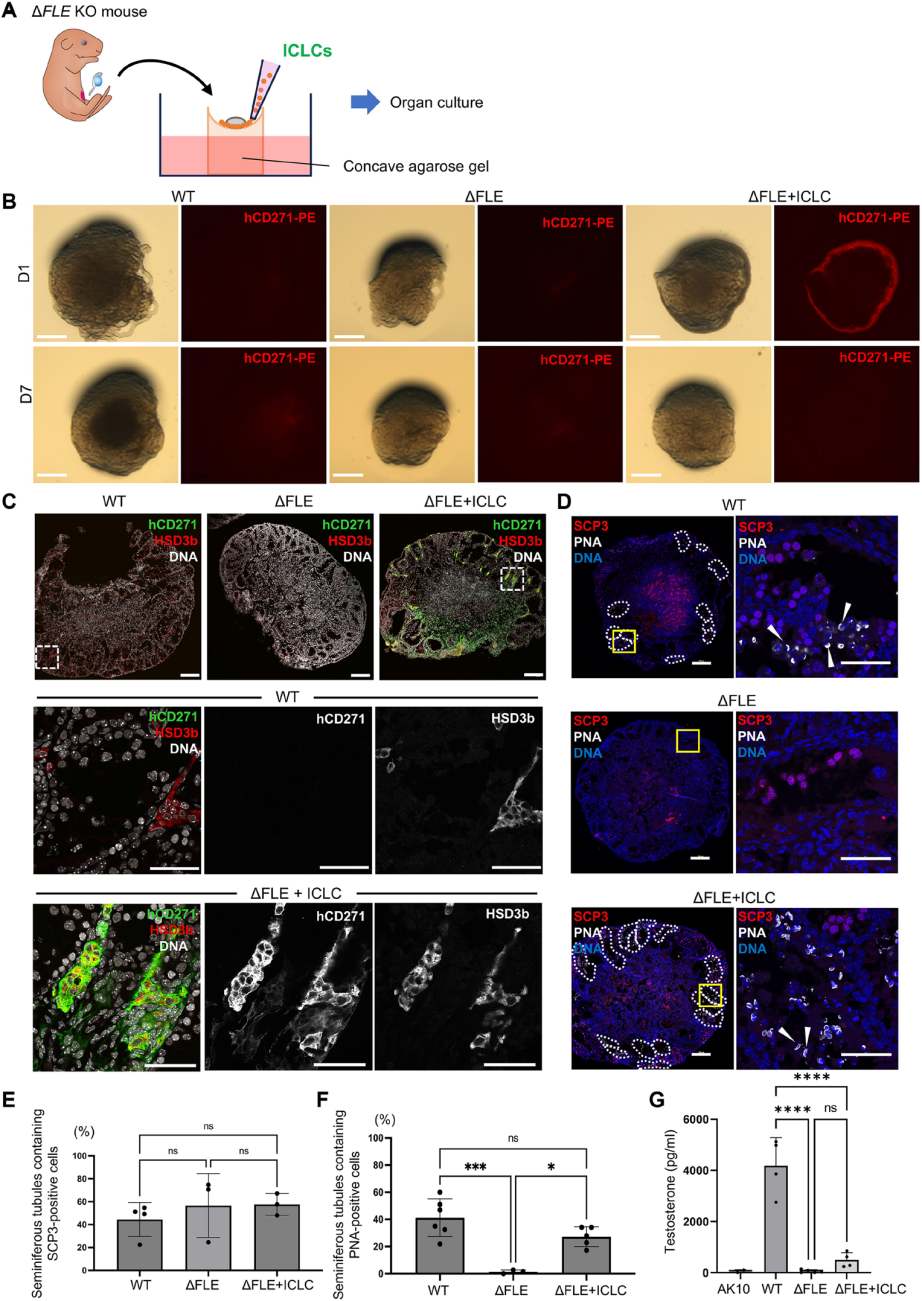
Functional characterization of ICLCs. (**A**) Schematic of the ICLC supplementation assay. (**B**) Representative images of cultured testis tissues from WT, Δ*FLE*, and Δ*FLE* mice supplemented with ICLCs (Δ*FLE* + ICLC). In the Δ*FLE* + ICLC group, ICLCs labeled with anti–hCD271-PE settled around the testis tissue within the concave agarose gel on D1. By D7, the accumulated ICLCs were no longer visible, suggesting their migration into the tissue along with photobleaching of PE. Scale bars, 500 μm. (**C**) Immunofluorescence images of testis tissue after 35 days of culture, stained with anti-hCD271, anti-HSD3b, and Hoechst. The boxed area in the WT panel (top row) is enlarged in the middle row, showing the merged image (left) and the individual channels for hCD271 and HSD3b (center and right). The boxed area in the Δ*FLE* + ICLC panel is enlarged in the bottom row in the same manner. Scale bars, 200 μm (top panels) and 50 μm (middle and bottom panels). (**D**) Immunofluorescence images of testis tissues (WT, Δ*FLE*, and Δ*FLE* + ICLC) after D35, stained with anti-SCP3, PNA, and Hoechst. Enlarged views of the yellow boxed areas in the left panels are shown on the right. Dotted lines mark seminiferous tubules containing PNA-positive cells. Scale bars, 200 μm (left panels) and 50 μm (right panels). (**E**) Bar graph showing the mean percentages (±SD) of seminiferous tubules containing SCP3-positive cells across the experimental groups. Statistical significance was assessed using Tukey’s test. ns, not significant. (**F**) Bar graph showing the mean percentages (±SD) of seminiferous tubules containing PNA-positive cells across the experimental groups. Statistical significance was assessed using Tukey’s test. *P < 0.05 and ***P < 0.001. (**G**) Testosterone concentrations measured in the culture medium during the D28-to-D35 period. AK10 indicates fresh (uncultured) medium. Tukey’s test was performed among all samples, excluding AK10. ****P < 0.0001.

To assess the functional properties of fTeSLCs, we first focused on interstitial cell–like cells (ICLCs), which were isolated via FACS as *hCD271^low^/PDGFR*α^+^ cells (fig. S13). To evaluate their role, we used Δ*FLE* mice, in which the fetal Leydig cell enhancer (FLE) of the *Nr5a1* gene is knocked out. These mice exhibit impaired differentiation and deficiency of both fetal and adult Leydig cells, leading to infertility with spermatogenesis arrested at the meiotic stage (*34*). Given this phenotype, we investigated whether ICLC supplementation into Δ*FLE* testicular tissue could rescue spermatogenesis under in vitro conditions. Testicular fragments from neonatal Δ*FLE* (6.5 to 8.5 days old) and WT littermates were cultured. ICLCs, labeled with a phycoerythrin (PE)–conjugated anti-hCD271 antibody and isolated from fTeSLCs at induction D8 and D9 (fig. S13), were added to Δ*FLE* fragments for organ culture (Fig. 4A). On D1, PE-labeled ICLCs were observed around the Δ*FLE* fragments but were no longer visible by D7 (Fig. 4B). This disappearance was primarily attributable to photobleaching of the PE signal, although hCD271-positive ICLCs had migrated into the tissue, and some had differentiated into HSD3b-positive Leydig cells (fig. S14). To examine their fate, testicular tissues were collected on D35 and subjected to immunostaining. In Δ*FLE* + ICLC samples, hCD271 staining confirmed the presence of ICLCs that had localized within the interstitial region (Fig. 4C). In WT testes, HSD3b1-positive cells, corresponding to endogenous Leydig cells, were detected in the interstitium, whereas, as expected, Δ*FLE* samples lacked HSD3b1-positive cells. Notably, in Δ*FLE* + ICLC fragments, many hCD271-positive cells coexpressed HSD3b1, indicating that a substantial proportion of ICLCs had differentiated into Leydig cells (Fig. 4C). To monitor the progression of spermatogenesis, we performed immunostaining for synaptonemal complex protein 3 (SCP3), a marker of spermatocytes, and peanut agglutinin (PNA), a marker of spermatids (Fig. 4D). WT testicular fragments contained both SCP3- and PNA-positive cells in the seminiferous tubules. As expected, Δ*FLE* testicular fragments contained SCP3-positive cells but lacked PNA-positive cells, indicating spermatogenic arrest at the meiotic stage (*34*). Notably, supplementation with ICLCs induced the appearance of PNA-positive cells, demonstrating that spermatogenesis progressed beyond meiosis to the formation of elongating spermatids. Quantitative analysis revealed no significant differences in the proportion of seminiferous tubules containing SCP3-positive cells among the three groups (Fig. 4E). However, ~30% of seminiferous tubules in Δ*FLE* + ICLC testicular fragments contained PNA-positive cells, a proportion comparable to that in WT testicular fragments (Fig. 4F). These results indicate that ICLC supplementation promotes spermatogenesis beyond meiosis and supports the formation of spermatids. To further characterize the functional capacity of ICLCs within the testicular environment, we measured testosterone levels in culture medium collected during the fourth week of culture (Fig. 4G). While testosterone levels in Δ*FLE* + ICLC testicular fragments were lower than those in WT fragments, they were still measurable and tended to be higher than those in Δ*FLE* fragments, although this did not reach statistical significance. These findings demonstrate that mouse ESC-derived ICLCs can differentiate into Leydig cells, produce testosterone, and support spermatogenesis.

To assess the fertility of the haploid cells, specifically round spermatids, we cultured Δ*FLE* + ICLC testicular fragments for 28 days and harvested round spermatids. Round spermatid injection (ROSI) was then performed, yielding 77 two-cell embryos. Notably, transfer of these embryos into six pseudopregnant females resulted in the birth of one healthy offspring (fig. S15 and table S1), providing clear evidence that ESC-derived ICLCs can ultimately support the generation of fertile gametes in vitro.

### SerLCs in fTeSLCs support spermatogenesis

To determine whether SerLCs found in fTeSLCs are functionally capable of supporting spermatogenesis, we used the in vitro Sertoli cell replacement method (*35*). This method involves transplanting donor Sertoli cells into the testes of *Amh*-diphtheria toxin receptor (*Amh*-DTR) Tg mice, which allow for selective elimination of endogenous Sertoli cells upon diphtheria toxin (DT) treatment.

As DT is highly potent, Sertoli cells in *Amh*-DTR mice are completely ablated within 4 to 7 days, leading to the subsequent death of germ cells because of the loss of their supportive niche. However, previous studies have shown that if the seminiferous epithelium is reconstituted with donor Sertoli cells, the remaining germ cells in the recipient can be maintained, allowing spermatogenesis to resume (*35*). For the present study, we performed a stepwise reevaluation of the DT concentration and confirmed that 5 ng/ml was sufficient to reliably ablate Sertoli cells (fig. S16, A to C).

**Fig. 5.**
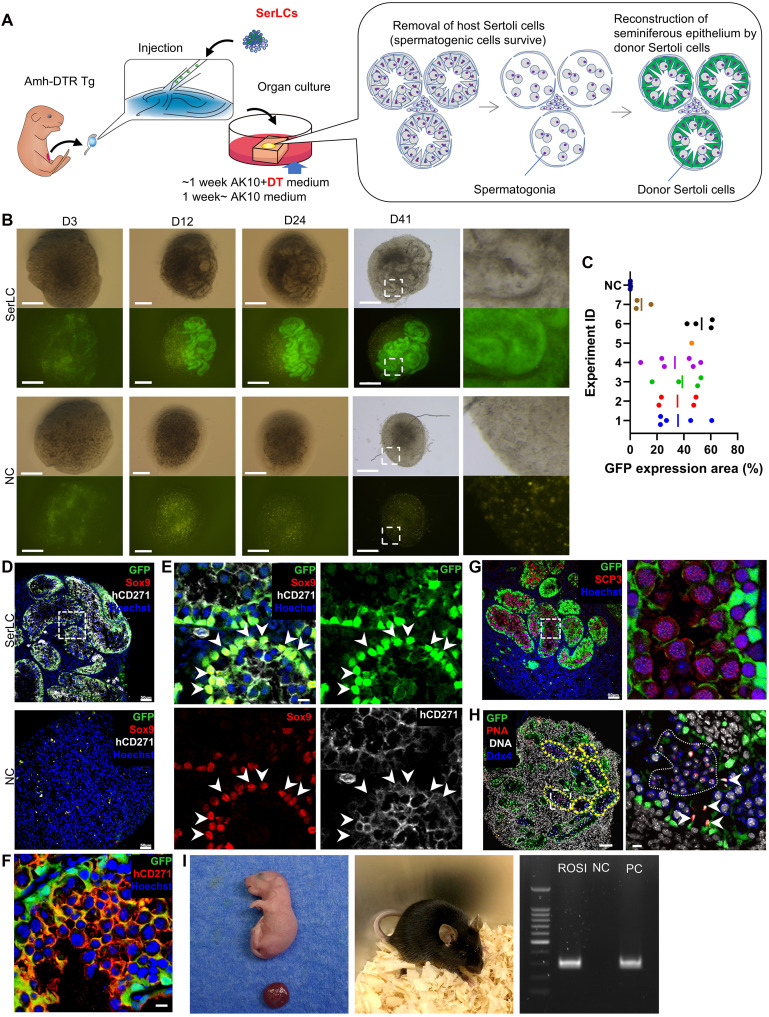
Functional characterization of SerLCs. (**A**) Schematic of the Sertoli cell replacement assay. (**B**) Time-lapse imaging of testes from DT-treated *Amh-*DTR mice injected with SerLC or noninjected NC tissues. Right panels show magnified views. Scale bars, 500 μm. (**C**) Quantification of CGFP-positive reconstructed seminiferous tubule area as a proportion of total tissue area after 3 to 5 weeks of culture. Data are from 31 testes across seven independent experiments. (**D**) Immunofluorescence image of reconstructed testes on D41 stained for GFP, Sox9, hCD271, and Hoechst. SerLC-injected tissues formed organized tubules, unlike NC tissues. Scale bars, 50 μm. (**E**) Higher magnification of the boxed region in (D). Sox9-positive SerLCs align along the basement membrane (arrowheads). Scale bars, 10 μm. (**F**) Morphology of SerLCs in D35 reconstructed tubules, stained for GFP, CD271, and Hoechst. Scale bar, 10 μm. (**G**) Immunofluorescence image on D35 showing SCP3-positive spermatocytes within GFP-positive tubules. Right: Magnified view of the boxed area. Scale bar, 50 μm. (**H**) Immunofluorescence image on D40 showing spermatids (PNA) and germ cells (Ddx4). Tubules containing spermatids are marked (yellow dotted lines). The magnified view shows round spermatids (dotted circle) and elongating spermatids (arrowheads). Scale bars, 100 μm (left) and 10 μm (right). (**I**) Offspring produced via ROSI with round spermatids from D48 reconstituted tubules. Left: Newborn pup. Center: Same mouse at a later stage. Right: Genotyping PCR for the *Amh-*DTR transgene confirms the offspring’s origin. Lanes: 100-bp ladder, NC, and PC (*Amh-*DTR gDNA).

The SerLCs were isolated using a two-gate cell sorting method: PDGFRα-negative and hCD271^high^/*Sox9*-CGFP^high^ fractions were collected as SerLCs (fig. S13). These cells were then injected into the seminiferous tubules of *Amh*-DTR Tg mouse testes, followed by in vitro culture of the testicular fragments in DT-containing medium (Fig. 5A). Consistent with our previous findings (*35*), DT treatment in negative control (NC) testicular fragments, where no SerLCs were injected, resulted in Sertoli cell ablation, complete loss of the seminiferous tubule structure, and germ cell depletion (Fig. 5B). Moreover, even after extended culture, no generation of seminiferous tubules was observed, as previously reported (*35*). In contrast, in testicular fragments where SerLCs were injected, the seminiferous epithelium structures were successfully reconstituted by the transplanted SerLCs, which expressed *Sox9*-CGFP (Fig. 5, B and C). In some tubules, CGFP-negative reticular regions were observed, suggesting the presence of germ cells (Fig. 5B). In contrast, NC tissues rapidly lost their tubular architecture and became unstructured aggregates (Fig. 5B). Cryosections of these tissues were subjected to immunostaining for GFP, hCD271, and Sox9 to confirm the integration and identity of transplanted SerLCs. The analysis revealed that the reconstructed seminiferous epithelium was composed of SerLCs coexpressing GFP and hCD271 (Fig. 5D). These cells also expressed endogenous Sox9 and exhibited morphological features typical of Sertoli cells, resembling those found in native seminiferous tubules (Fig. 5, D and E). Specifically, their cytoplasm extended toward the lumen of the tubules, and their nuclei were properly aligned near the basement membrane (Fig. 5, E and F) (*36*). In contrast, NC testicular fragments showed no seminiferous tubule structures, Sertoli cells, or evidence of spermatogenesis (Fig. 5D), consistent with previous reports (*35*).

To assess whether the reconstructed seminiferous epithelium could support spermatogenesis, we performed immunostaining for SCP3 and PNA (Fig. 5, G and H). Notably, a large number of spermatocytes with SCP3-positive chromosomal configurations were observed exclusively in the reconstructed, GFP-expressing tubules and were absent in the unreconstructed regions (Fig. 5G).

In addition, round and elongating spermatids positive for PNA (Fig. 5H and fig. S17, A and B) and for the elongating spermatid marker Tnp1 (transition protein 1; fig. S17C) (*37*) were detected, indicating successful progression through meiosis and spermatid differentiation. In these cultures, Prm1 (protamine 1) expression was not evident in spermatids, which aligns with their identification as elongating spermatids.

Immunostaining of cryosections from SerLC-engrafted testicular tissues (defined as ≥40% area occupied by SerLCs) further confirmed the presence of haploid cells in 12 of 14 analyzed samples (from seven independent experiments) (table S2). These results indicate that SerLCs effectively repopulated the seminiferous epithelium and functionally supported host spermatogenesis.

Last, to assess whether the generated haploid cells were functionally competent, we performed ROSI. This procedure resulted in the birth of one female offspring (Fig. 5I and table S3). The offspring developed normally and, upon mating with a WT male, produced healthy progeny of its own, thereby demonstrating full reproductive competence (fig. S18 and table S4). These findings confirm that SerLCs derived from fTeSLCs can replace endogenous Sertoli cells, support spermatogenesis, and generate haploid cells capable of transmitting fertility to the next generation.

## DISCUSSION

In the present study, we developed a culture system that directs XY ESCs toward differentiation into fTeSLCs. While recent studies have focused on deriving testicular somatic cells, particularly Sertoli cells, from pluripotent stem cells, generating fully functional cells capable of supporting spermatogenesis has remained a challenge. In our research, we successfully isolated ICLCs and SerLCs from fTeSLCs and evaluated their functional roles using interstitial supplementation and Sertoli cell replacement methods. ICLCs, when supplemented into the testicular interstitium of Δ*FLE* mice, differentiated into Leydig cells, produced testosterone, and ultimately rescued spermatogenic failure. These findings demonstrate that ICLCs can support spermatogenesis and further suggest their potential for treating infertility associated with Leydig cell dysfunction in the future. An important unresolved question, however, is the process by which Leydig cells differentiate from ICLCs. During in vivo development, it has been reported that fetal Leydig cells dedifferentiate and subsequently redifferentiate into adult Leydig cells (*34*). Therefore, further detailed studies are required to clarify whether a similar phenomenon occurs in our system.

Meanwhile, both the ICLC supplementation and SerLC-mediated seminiferous epithelium reconstruction experiments supported the progression of spermatogenesis to the haploid stage. Notably, one offspring was obtained from each of these experiments via ROSI using spermatids produced in the cultured testicular tissues. While these results demonstrate the ability of both ICLCs and SerLCs to support spermatogenesis, the very limited offspring yield indicates that further optimization of the organ culture system is required to improve efficiency. In the ICLC supplementation experiment, testosterone levels in the ICLC-supplemented group were lower than those in WT testes, which may have contributed to the reduced efficiency of spermatogenesis. This could be related to both the number of supplemented ICLCs and number and maturity of the Leydig cells differentiated from them. Therefore, optimization of these culture conditions will be necessary. Previous studies have similarly reported low offspring yields in complex approaches combining organ culture and transplantation (*38*, *39*), most likely due to both the inherently low efficiency of spermatogenesis in organ culture and limited engraftment of transplanted cells. In conventional organ culture, which more efficiently induces spermatogenesis, the live birth rate after ROSI was 43.8% (*40*). In contrast, when an additional transplantation step was included, the reported rates dropped to 12.5% (*39*) and 5% (*38*). Furthermore, as in those studies, intracytoplasmic sperm injection could not be performed in the present study, because mature spermatozoa were not found in the cultured tissues. Even though a few elongating spermatids were observed, they remained tightly associated with Sertoli cells, preventing their isolation for use in microinsemination. Thus, future improvements in both transplantation efficiency and spermatogenesis induction will be necessary to enhance the robustness of this system.

Using the same method previously established for inducing FOSCL from XX ESCs (*13*), we initially expected that applying it to XY ESCs would yield the male counterpart. It was therefore unexpected to observe that the cells induced from XY ESC cells were female in character, as evidenced by their high expression of *Foxl2*. This suggests that *Sry* expression may have been insufficient during the ESC cultivation and was therefore unable to drive male gonadal differentiation. On the other hand, given that the Wnt pathway is known to promote feminization, we considered that addition of CHIR, a Wnt pathway activator, to the D0 and D1 media may have had a strong and prolonged effect, persistent beyond the intended period despite two complete medium changes at D2 and D4. To mitigate this potential issue, we introduced IWR1, a Wnt inhibitor, into the D4 medium, which effectively suppressed feminization and redirected the cells toward masculinization, resulting in the generation of functional SerLCs. Further optimization of the masculinization protocol will be necessary to enhance the efficiency of fTeSLC generation from XY ESCs.

scRNA-seq analysis revealed that fTeSLCs contained most cell types that constitute the fetal testis, including Sertoli cells and stromal/interstitial cells. Notably, fTeSLCs also included supporting-like lineage cells, which originate from progenitor cells at an earlier stage than Sertoli cells and eventually contribute to the formation of the rete testis (*33*). This suggests that our differentiation protocol successfully recapitulates key aspects of in vivo gonadogenesis. Given these findings, our differentiation induction method could serve as a valuable tool for studying gonadal sex differentiation and testicular formation during embryonic development.

Another challenge remains: We have yet to successfully reconstruct testicular organoids from fTeSLCs, in contrast to the in vitro reconstruction of ovarian tissue from FOSLCs alone, which successfully induced oogenesis. In a previous study, we demonstrated testicular tissue reconstruction by culturing aggregates of isolated neonatal testicular cells at the gas-liquid interface (*41*). However, this method was incomplete, as spermatogenesis was inconsistent and largely stalled at the meiotic phase.

In the present study, we focused specifically on interstitial cells and Sertoli cells in fTeSLCs, which play a crucial role in supporting spermatogenesis, and successfully validated their functional competency of each cell type individually. Moving forward, improving testicular tissue reconstruction techniques using fTeSLCs to generate fully reconstituted testicular tissue will not only further elucidate their functional properties but also open new avenues for therapeutic applications and reproductive research.

## MATERIALS AND METHODS

### Animals

*Amh*-DTR Tg (strain: ICR, C57BL/6, and their mixture) (*42*), ICR (CLEA Japan), and Δ*FLE* (*34*) mice were used. Animals were fed ad libitum with MF hard pellets (Oriental Yeast Co., Tokyo). Drinking water was acidified to pH 2.8 to 3.0 by HCl. All animal experiments were performed in accordance with the Guidelines for Proper Conduct of Animal Experiments (Science Council of Japan) and were approved by the Institutional Committee of Laboratory Animal Experimentation (Animal Research Center of Yokohama City University; protocol no. F-A-23-025).

### Construction of the targeting vector and guide RNA

For construction of the targeting vector for *Sox9-2A-CGFP*, the 869– and 807–bp (base pair) genomic fragments of the Sox9 gene were amplified from genomic DNA of a C57Bl/6J mouse by PCR and used for the left and right arms, respectively. CGFP was cloned from *pcDNA3-Clover* (Addgene; no. 40259) by PCR using primers with a P2A peptide sequence added to the 5′ terminus of the CGFP gene. After cleavage at Kpn I and Sac I sites to replace the Rosa homology arm of the *pDonor-Rosa26* plasmid (Addgene; no. 37200) and *Sox9-2A-CGFP*, each cloned DNA fragment was joined together using an In-Fusion HD cloning kit (Takara Bio, Shiga, Japan) to generate the *Sox9-CGFP* targeting vector.

A Cas9 and chimeric guide RNA expression plasmid, *pX458* (*pSpCas9-2A-GFP*) (Addgene; no. 48138), which expresses Cas9 nuclease, single guide RNA, and GFP, was obtained from Addgene (no. 42335). The guide RNAs targeting Sox9 genes (CCCTGAGAAGAGAAAAGCTA) were ligated into Bbs I sites in the plasmid.

*Nr5a1-*hCD271 ESCs [embryos obtained from crosses between 129X1/SvJ females and *Nr5a1*-hCD271 (C57BL/6J) males] were harvested using TrypLE Express (no. 12604021, Thermo Fisher Scientific, Waltham, MA) and resuspended in a Mouse ES Cell Nucleofector solution (no. VPH-1001, Lonza, Basel, Switzerland) for electroporation with 8 μg of targeting vector plasmids and 4 μg of CRISPR-Cas9–expressing plasmid using a Nucleofector 2b electroporation system with program A-030. After electroporation, ESCs were cultured on mouse embryonic fibroblast (MEF) feeders in N2B27 medium for 1 day, and then cells transiently expressing GFP of the *pX458* vector were sorted by FACS, followed by culturing on a MEF feeder. After about a week, the emerged colonies were picked up with a P200 pipette; half the colonies were cryopreserved using a STEM-CELLBANKER freezing solution (no. CB047, Takara Bio), and the remaining half were genotyped by PCR. The clones having a knockin of CGFP were thawed and propagated for the induction experiment. They were designated N271S9C ESCs.

### Production of the chimeric mouse

Fertilized eggs were harvested from superovulated ICR mice, and five to eight ESCs were carefully injected into eight-cell stage embryos using micromanipulators. After injection, embryos were cultured in KSOM medium until the blastocyst stage and then transferred into the uteri of pseudopregnant ICR foster mothers.

### fTeSLC induction from ESCs

The ESCs used in the fTeSLC induction experiments were N271S9C ESCs, as explained above. They were maintained on MEF feeders for regular proliferation and passage but were cultured in a laminin-coated plate without feeder cells for two passages before use in for induction experiments. The derivation of fTeSLCs from ESCs was performed in the manner previously reported for the induction of FOSLCs, with some modifications (*13*). Induction of EpiLCs from ESCs followed a previous paper (*43*). Briefly, ESCs were cultured with N2B27 medium containing 1% KSR (no. 10828028, Gibco), FGF2 (12 ng/ml; no. 13256-029, Gibco), and activin A (20 ng/ml; no. 18585-94, Nacalai Tesque) for 42 hours. Then, 3 × 10^6^ EpiLCs dissociated with TrypLE were seeded in a 35-mm dish named EZSPHERE SP (type 904; AGC Techno Glass, Shizuoka, Japan) and cultured in GK15 medium [Glasgow’s minimum essential medium supplemented with 15% KSR, 0.1 mM nonessential amino acids, 1 mM sodium pyruvate, 0.1 mM 2-mercaptoethanol, 1× antibiotic-antibiotic, and 2 mM l-glutamine (all Thermo Fisher Scientific)] containing 14 μM CHIR (no. 038-23101, FUJIFILM Wako Pure Chemical, Japan), BMP4 (1 ng/ml; no. 314-BP-010, R&D Systems, Minneapolis, MN), and epidermal growth factor (50 ng/ml; no. 2028-EG-200, R&D Systems) to form cell aggregations. The next day (D1), the cell aggregates were collected and suspended in medium containing 2% FCeM, a cell suspension polymer solution (Nissan Chemical, Tokyo), and transferred to a low-cell-binding culture dish (no. 628979, Greiner Bio-one, Frickenhausen, Germany) for further cultivation. On D2, the culture medium was changed to GK15 + 2% FCeM containing 3 μM retinoic acid (no. R2625, Sigma-Aldrich, St. Louis, MO), Sonic hedgehog (30 ng/ml; no. 461-SH, R&D Systems), 1 μM PD (no. 162-25291, FUJIFILM Wako Pure Chemical), epidermal growth factor (50 ng/ml), and BMP4 medium (1 ng/ml). After 49 hours of culture, on D4, the medium was replaced with GK15 + 2% FCeM containing BMP4 (20 ng/ml), FGF9 (2 ng/ml; no. 100-23, PeproTech, NJ, US), and 20 μM IWR1 (no. 037-25131, FUJIFILM Wako Pure Chemical) medium. Some experiments were performed using GK10 medium with a 10% concentration of KSR and conventional aggregation culture method (*13*) using low-cell-binding culture 96-well plates (no. 650979, Greiner Bio-one) without FCeM. The culture incubator was supplied with 5% carbon dioxide in air and maintained at 37°C.

### Establishment of an *Amh*-DTR ESC line

To establish the ESC line, blastocysts were derived from *Amh*-DTR intercrosses and cultured following a previously published protocol (*44*). The cells were maintained in a medium composed of Dulbecco’s modified Eagle’s medium with the following supplements: 15% KSR, leukemia inhibitory factor (1000 IU/ml; no. ESG1107, ESGRO), 1 μM PD, 3 μM CHIR, 1 mM sodium pyruvate, 1× GlutaMAX, 1× minimum essential medium nonessential amino acids, and 110 μM 2-mercaptoethanol.

### Flow cytometric analysis and cell sorting

Collected cell aggregations were digested in a 0.05% trypsin-EDTA solution (Thermo Fisher Scientific) at 37°C for 8 min with shaking. After passing through a cell strainer with a 40-μm pore size (Becton Dickinson, Franklin Lakes, NJ), the isolated cells were suspended in FACS buffer (0.5% bovine serum albumin in phosphate-buffered saline). Anti–CD140a (PDGFRα)-APC (allophecocyanin) (1:250; no. 135905, BioLegend, CA, US) and anti-human CD271-PE (1:50; no. 557196, Becton Dickinson) were added to the cell suspension and incubated for 15 min on ice. After washing once in the FACS buffer, the cells were resuspended in the FACS buffer. For flow cytometric analysis and cell sorting, Guava EasyCyte (Merck Millipore, Burlington, MA) and Sony MA900 (Sony, Tokyo) were used, respectively. The sorting was performed in “Yield mode” to prioritize cell recovery, and when these samples were reanalyzed by FCM, the purity ranged from 91 to 97%.

### Magnetic-activated cell sorting

To isolate cells, cell aggregates cultured until D6 were dissociated into a single-cell suspension using a method similar to that for FCM sample preparation. For purification of Nr5a1-hCD271–positive cells, the dissociated cells were labeled with hCD271 Microbeads for 15 min and passed through an LS column according to the MACSelect LNGFR MicroBeads (Miltenyi Biotec, no. 130-091-330) protocol.

### qPCR analysis

Cells were lysed with a SuperPrep II Cell Lysis & RT Kit (Toyobo, Osaka, Japan, no. SCQ-401), and cDNA synthesis was performed from the lysate. qPCR analysis was performed with Power SYBR Green Master Mix (Applied Biosystems, Foster City, CA, no. A66732) on a StepOnePlus real-time qPCR system (Applied Biosystems). Gene expression levels were calculated as ΔCt normalized by the mean Ct (cycle threshold) value of β-actin. The primers used in this study are shown in table S5.

### Observations and immunohistochemistry

The cultured tissues were observed under a stereomicroscope equipped with an excitation light for GFP (M205 FA; Leica, Wetzlar, Germany). Fluorescence histochemical staining on testis cryosections (7 μm thick) was performed as previously described (*45*). For immunocytochemistry, harvested cells were suspended in phosphate-buffered saline, applied to adhesive-coated glass slides (Matsunami Glass Ind., Ltd., no. CRE-03), and incubated for 20 min to allow for cell attachment. Subsequently, the cells were fixed with 4% paraformaldehyde for 20 min. Staining was then performed using the same protocol as for the fluorescence histochemical staining. The following antibodies were used as the primary antibody: mouse anti-hCD271 antibody (1:1000; no. 557194, BD), mouse anti–hCD271-Alexa 647 antibody (1:50; no. 345113, BioLegend), mouse anti-Nr2f2 antibody (1:100; no. PP-H7147-00, R&D), rabbit anti-Gata4 antibody (1:50; no. sc-9053, Santa Cruz), rabbit anti-Pax8 (1:1000; no. 10336-1-AP, Proteintech), mouse anti-SCP3 antibody (1:100; no. ab97672, Abcam), rabbit anti-Ddx4 antibody (1:400; no. ab13840, Abcam), rabbit anti-Sox9 antibody (1:200; no. KO608, TransGenic), rat anti-Nr5a1 antibody (1:100; no. KO610, TransGenic), goat anti-Amh antibody (1:200; no. sc-6886, Santa Cruz), chicken anti-GFP antibody (1:1000; no. ab13970, Abcam), rat anti-Tra98 antibody (1:1000; Bioacademia), rabbit anti-HSD3b antibody (1:200; no. KO607, TransGenic), rabbit anti-Tnp1 antibody (1:200; no. 17178-1-AP, Proteintech), and mouse anti-Prm1 antibody (1:200; no. MAb-Hup1N-150, Briar Patch Bioscience). The secondary antibodies used were goat anti-mouse immunoglobulin G (IgG), goat anti-chicken IgG, goat anti-rabbit IgG, and goat anti-rat IgG, conjugated to Alexa 488, Alexa 555, or Alexa 647 (1:200; Invitrogen). Alexa 568– or Alexa 647–conjugated PNA was used to detect acrosomes (1:400; nos. L32458 and L32460, Invitrogen). Nuclei were counterstained with Hoechst 33342 dye (no. 346-07951, Dojindo, Kumamoto, Japan). Specimens were observed with a confocal laser microscope (FV-1000D from Olympus, Tokyo, or AX from Nikon, Tokyo).

### scRNA-seq library construction and sequencing

For scRNA-seq, Nr5a1-positive cells enriched via MACS were used as the ESC-derived cell sample. The E10.5 samples included dorsal mesenchymal tissue surrounding the gonads. For the E11.5 and E12.5 samples, the gonads were collected after the removal of the mesonephric tissue. Library construction was performed on ESC-derived cells and embryonic gonadal cells using Chromium Single Cell 3′ version 3 and version 2 Reagent Kits (10x Genomics), respectively, as previously reported (*13*). Briefly, the dissociated cells were mixed with Single Cell Master Mix to generate gel-based emulsions. RNA from each gel-based emulsion was reverse transcribed, and the resulting cDNA was amplified by PCR with the primers in the kit. Sequencing libraries suitable for Illumina sequencer were prepared from the amplified cDNA. The scRNA-seq libraries were sequenced with paired-end reads on the Illumina HiSeq X Ten platform.

### Preprocessing of scRNA-seq data

The raw paired-end sequence data were converted to FASTQ format files and mapped to the mouse mm10 genome using Cell Ranger software version 3.0.2 (10x Genomics). The output from Cell Ranger was processed using the Seurat R package (*46*). The data were loaded, and a unique molecular identifier count matrix was generated using the Read10X function. Low-quality cells and data from duplicates were filtered out by the subset function with the following parameters, which were empirically decided on the basis of the distribution of each parameter in a sample: XX_E10.5: nFeature_RNA > 1000 & nFeature_RNA < 4000 & percent.mt < 12; XX_E11.5: nFeature_RNA > 2000 & nFeature_RNA < 6000 & percent.mt < 10; XX_E12.5: nFeature_RNA > 2000 & nFeature_RNA < 5000 & percent.mt < 10; XX_FOSLCs: nFeature_RNA > 1500 & nFeature_RNA < 4000 & percent.mt < 12; XY_E10.5: nFeature_RNA > 1000 & nFeature_RNA < 4000 & percent.mt < 10; XY_E11.5: nFeature_RNA > 500 & nFeature_RNA < 5000 & percent.mt < 20; XY_E12.5: nFeature_RNA > 2000 & nFeature_RNA < 5000 & percent.mt < 8; XY_fTeSLCs: nFeature_RNA > 1000 & nFeature_RNA < 4000 & percent.mt < 12. For the retained cells, the molecular counts of a gene in a cell were divided by the total molecular counts for that cell, multiplied by 10,000 for normalization, and transformed into the natural logarithm using the log1p function with the NormalizeData function. All data were then integrated using the precalculated anchor sets calculated using the FindIntegrationAnchors function. To alleviate the impact of cell cycle heterogeneity, cell cycle phase scores were calculated using the CellCycleScoring function with normal cell cycle markers (*47*), and the cell cycle phase differences were regressed out using the ScaleData function.

### Clustering and dimension reduction

The integrated data of all samples were scaled (mean expression: 0; variance: 1), and then Seurat package’s RunPCA function was used to perform principal components analysis (PCA) followed by graph-based clustering using the FindNeighbors and FindClusters functions in the space of PC1 to PC10 of PCA with a resolution of 0.2 or in the space of PC1 to PC10 of PCA with a resolution of 0.3 for the whole-cell population or GSC population analyses, respectively. Dimensional reduction was performed using UMAP with the RunUMAP function.

### Identification of cluster-specific genes

Each cluster-specific gene was identified using the FindAllMarkers function with the parameters min.pct = 0.25 and logfc.threshold = 0.25, and the MAST algorithm implemented in the Seurat package (*48*) was used to identify the clusters. The top five cluster-specific genes were visualized as a heatmap using the DoHeatmap function.

### Correlation matrix

Male GSC clusters of E12.5 and fTeSLCs were assigned to Sertoli cells (clusters 1 and 6), progenitor cells (clusters 0, 2, and 3), and interstitial cells (clusters 4 and 5), resulting in six categories (XY_E12.5-Sertoli, XY_E12.5-Progenitor, XY_E12.5-Interstitial, XY_fTeSLCs-Sertoli, XY_E12.5-fTeSLCs, and XY_E12.5-fTeSLCs). Correlation coefficients for all combinations of the two categories were calculated using the average expression levels of each gene in all cells in the category. Hierarchical clustering was performed using the hclust and dist functions with the default settings, implemented in the stats R package. The correlation matrix and hierarchical clustering were visualized as a heatmap.

### ICLC supplementation experiment in Δ*FLE* mouse testes

ICLCs were isolated by FACS as the hCD271^low^/PDGFRα^+^ population and suspended in α-minimum essential medium (Thermo Fisher Scientific) supplemented with 10% KSR (AK10 medium). Testes were harvested from 6.5-day-old WT and Δ*FLE* mice, then decapsulated, bisected, and placed on agarose gel partially immersed in AK10 medium. To each Δ*FLE* testis, ~50 × 10^4^ ICLCs suspended in 10 μl of AK10 were added using a micropipette. Concave agarose gel was used to prevent cell loss resulting from runoff. The concave agarose gel was prepared by dissolving 1.5% (w/v) agarose in Milli-Q water, sterilizing by autoclaving, and dispensing 0.5 ml into each well of a 24-well plate. The concave shape formed naturally because of the meniscus during gel solidification. Testicular organ culture was performed as we previously described at 34°C in a 5% CO_2_ incubator (*40*). For the quantification of testosterone, culture medium was collected after 7 days of culture and stored at −80°C until use. The measurements were performed at ASKA Pharma Medical Co., Ltd. (Fujisawa, Japan), using liquid chromatography–tandem mass spectrometry.

### In vitro Sertoli cell replacement method

SerLCs were sorted by FACS and resuspended in AK10 medium at concentrations of 5 × 10^4^ to 15 × 10^4^ cells/μl. These single-cell suspensions were injected into the seminiferous tubules through the rete testis of *Amh*-DTR Tg mice at 3.5 to 10.5 days postpartum (dpp) as described previously (*39*, *49*). After injection, the testes were cut into pieces and cultured in AK10 medium containing DT (5 ng/ml) as previously described (*35*). One week later, the medium was changed to regular AK10 without DT. After 35 to 50 days, the tissues were fixed, and frozen sections were prepared. Medium change was performed once a week. The culture incubator was supplied with 5% CO_2_ in air and maintained at 34°C. The cultured tissues were observed or photographed weekly using a fluorescence stereomicroscope. After 5 to 7 weeks, the tissues were fixed, and cryosections were prepared.

### Measurement of transplanted SerLC colony formation area

Bright-field and GFP fluorescence images of cultured testicular tissues were captured using a fluorescence stereomicroscope (Leica, Wetzlar, Germany), and the colonization area was calculated as previously described (*35*). The images used for quantification were taken 3 to 5 weeks after transplantation when the engraftment of transplanted cells was complete. The total area of testicular tissue was measured by tracing the outline of the tissue using the freehand selection tool in National Institutes of Health ImageJ (version 1.54). The GFP-expressing area was measured by automatically selecting the green color of GFP using the Color Threshold function of ImageJ on fluorescence photographs.

### Round spermatid injection

Round spermatid injection was performed as previously described (*50*, *51*). The cultured testicular tissues were dissected under a stereomicroscope. Mature MII oocytes were collected from superovulated B6D2 females and were freed from cumulus cells by 0.5% bovine testicular hyaluronidase treatment. The oocytes were preactivated by Ca^2+^-free CZB medium containing 2.5 mM SrCl_2_. Round spermatids were identified by their round nucleus and high cytoplasm/nucleus ratio (*52*). These cells were collected and injected into the preactivated oocytes at telophase II (40 to 50 min after activation) using a piezo-driven micromanipulator. Fertilized oocytes were cultured for 24 hours, and two-cell embryos were transferred into the oviducts of pseudopregnant ICR females. A live fetus recovered on D19.5 was reared by a nursing ICR-foster mother.

### Genotyping

To extract genomic DNA, mouse ear pieces or ESCs were incubated in 50 mM NaOH at 96°C for 10 min and then neutralized with tris-HCl (pH 8.0). PCR was performed with a KOD-ONE kit (Toyobo) using this genomic DNA lysate as a template. The primers used in this study are shown in table S5.
