# Supplementary material for: Generation of functionally competent testicular somatic cells from pluripotent stem cells
Source: Sci Adv. 2026 Feb 26;12(9):eadz0269. doi: 10.1126/sciadv.adz0269 (PMC13250946; doi:10.1126/sciadv.adz0269)
Supplement: Supplementary file 1 — Figs. S1 to S18 Tables S1 to S5 [file sciadv.adz0269_sm.pdf]

Science Advances
AAAS

Supplementary Materials for

Generation of functionally competent testicular somatic cells from pluripotent stem cells

Takuya Sato *et al.*

Corresponding author: Katsuhiko Hayashi, hayashik@gcb.med.osaka-u.ac.jp;
Takehiko Ogawa, ogawa@yokohama-cu.ac.jp; Takuya Sato, tsato@yokohama-cu.ac.jp

Sci. Adv. **12**, eadz0269 (2026)
DOI: 10.1126/sciadv.adz0269

This PDF file includes:

Figs. S1 to S18
Tables S1 to S5

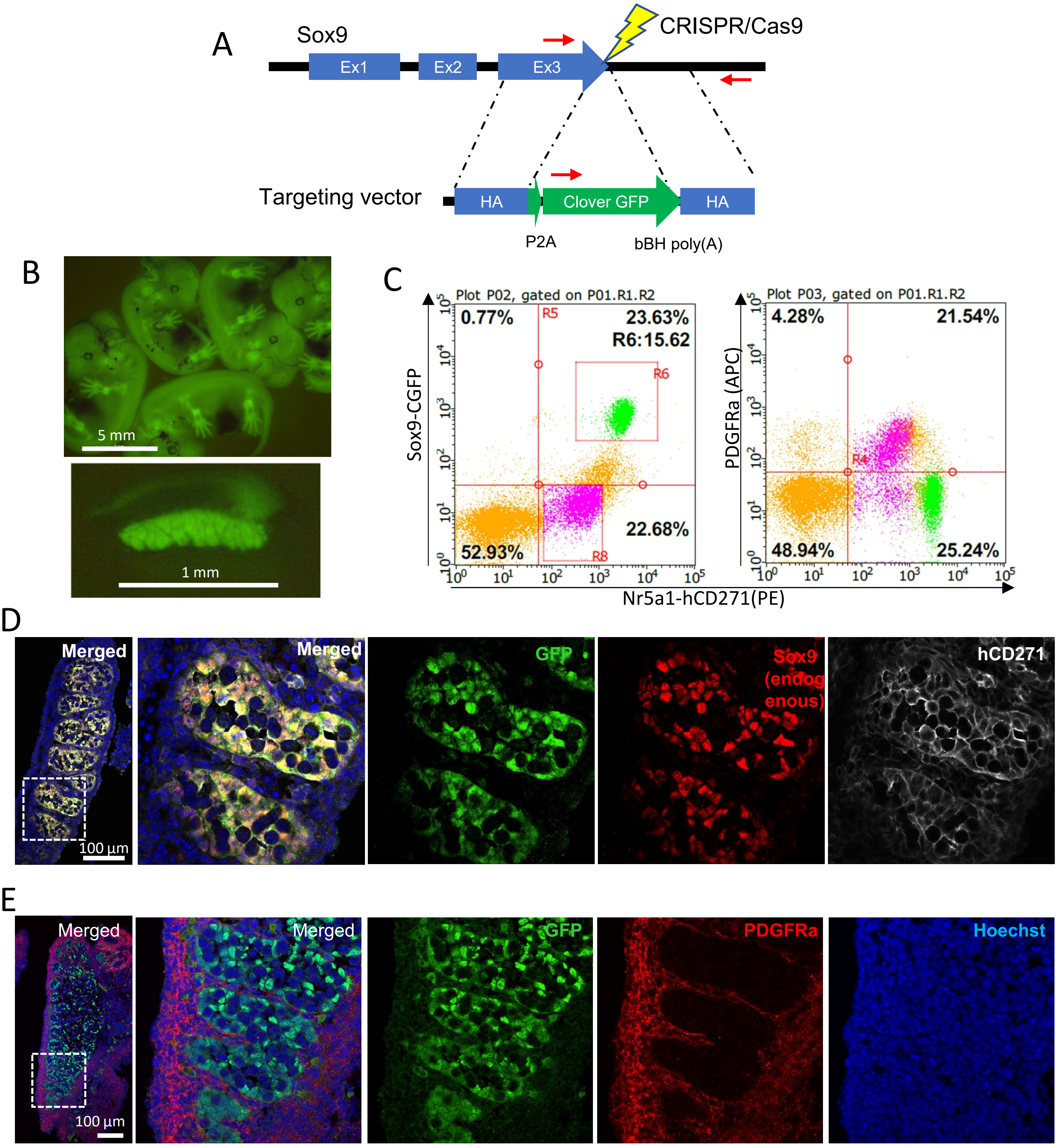

Figure S1. Generation of reporter ESCs and analysis of chimeric mice.
(A) Schematic representation of CGFP knock-in strategy at the *Sox9* locus in *Nr5a1-hCD271* ESCs. (B) Chimeric embryo generated with Nr5a1-hCD271/Sox9-CGFP ESCs. CGFP expression is observed in the whole body (upper) and testis (lower) of E12.5 chimeric embryo. (C) Flow cytometric analysis of testicular cells from E12 chimeric mice using anti-hCD271-PE and anti-PDGFRα-APC antibodies. Two distinct populations were identified: Nr5a1-hCD271^{high}/Sox9-CGFP^{high} cells (R6, light green), which were PDGFRα-negative and considered Sertoli cells; and Nr5a1-hCD271^{low}/Sox9-CGFP^{-} cells (R8, purple), which were PDGFRα-positive and classified as interstitial cells. (D) Immunostaining of cryosections from the testis of an E12.5 Nr5a1-hCD271/Sox9-CGFP embryo, an offspring of the chimera shown in (B), with antibodies against hCD271 (white), GFP (green) and Sox9 (red), counterstained with Hoechst (blue). Magnified views of the areas outlined by dotted lines are shown on the right. (E) Immunostaining of cryosections of E12.5 testis of Nr5a1-hCD271/Sox9-CGFP embryo with antibodies against GFP (green) and PDGFRa (red), counterstained with Hoechst (blue). Merged images are shown with their corresponding separate channels and magnified views of the areas outlined by dotted lines are presented on the right.

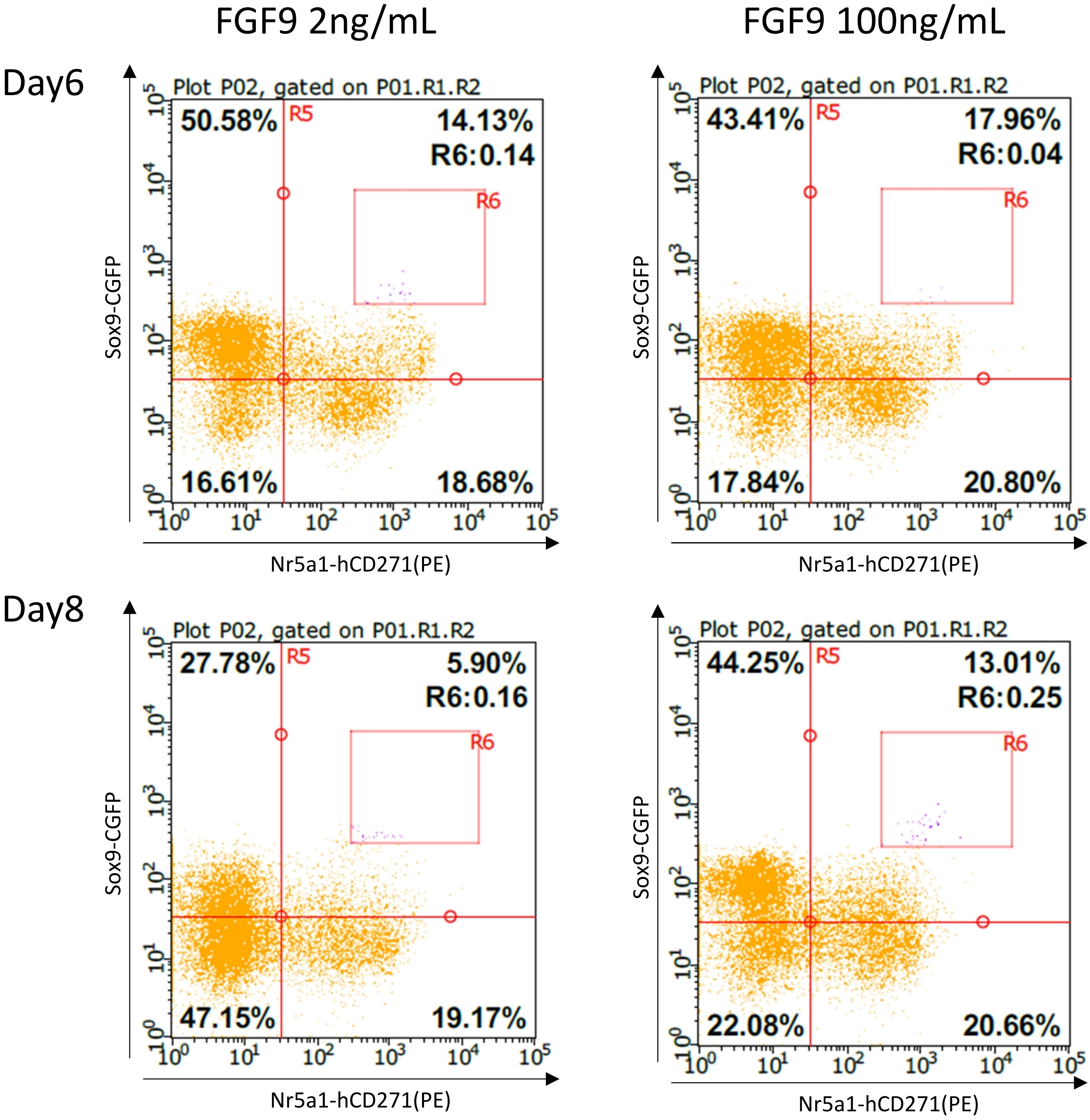

Figure S2. Increasing FGF9 concentration does not enhance Sertoli cell induction
Increasing FGF9 concentration from original 2 ng/mL to 100 ng/mL in the D4 medium had no significant effect on the induction of Nr5a^{high}/hCD271^{high}/Sox9-CGFP^{high} cells, i.e., Sertoli cells, on either day 6 or day 8.

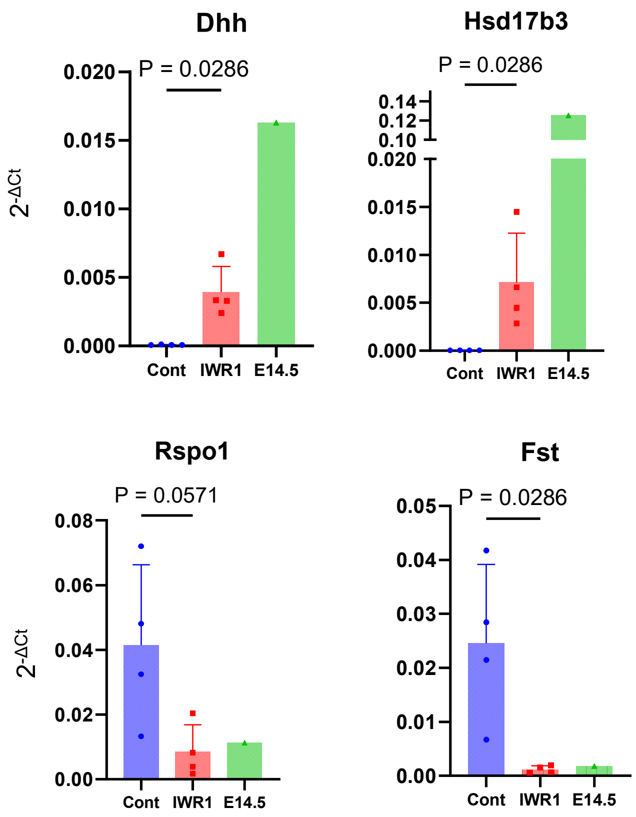

Figure S3. Expression of gonadal somatic cell marker genes and immunostaining analysis of induced Nr5a1-hCD271⁺ cells

RT-qPCR analysis of Nr5a1-hCD271⁺ cells sorted from cultures on D8. Expression levels of Dhh, Hsd17b3, Fst, and Rso1 were quantified. Relative expression is measured as $2^{-\Delta Ct}$ values, normalized against β-actin. Data represent the mean ± SD of four independent experiments, and significance was determined using the Mann-Whitney test.

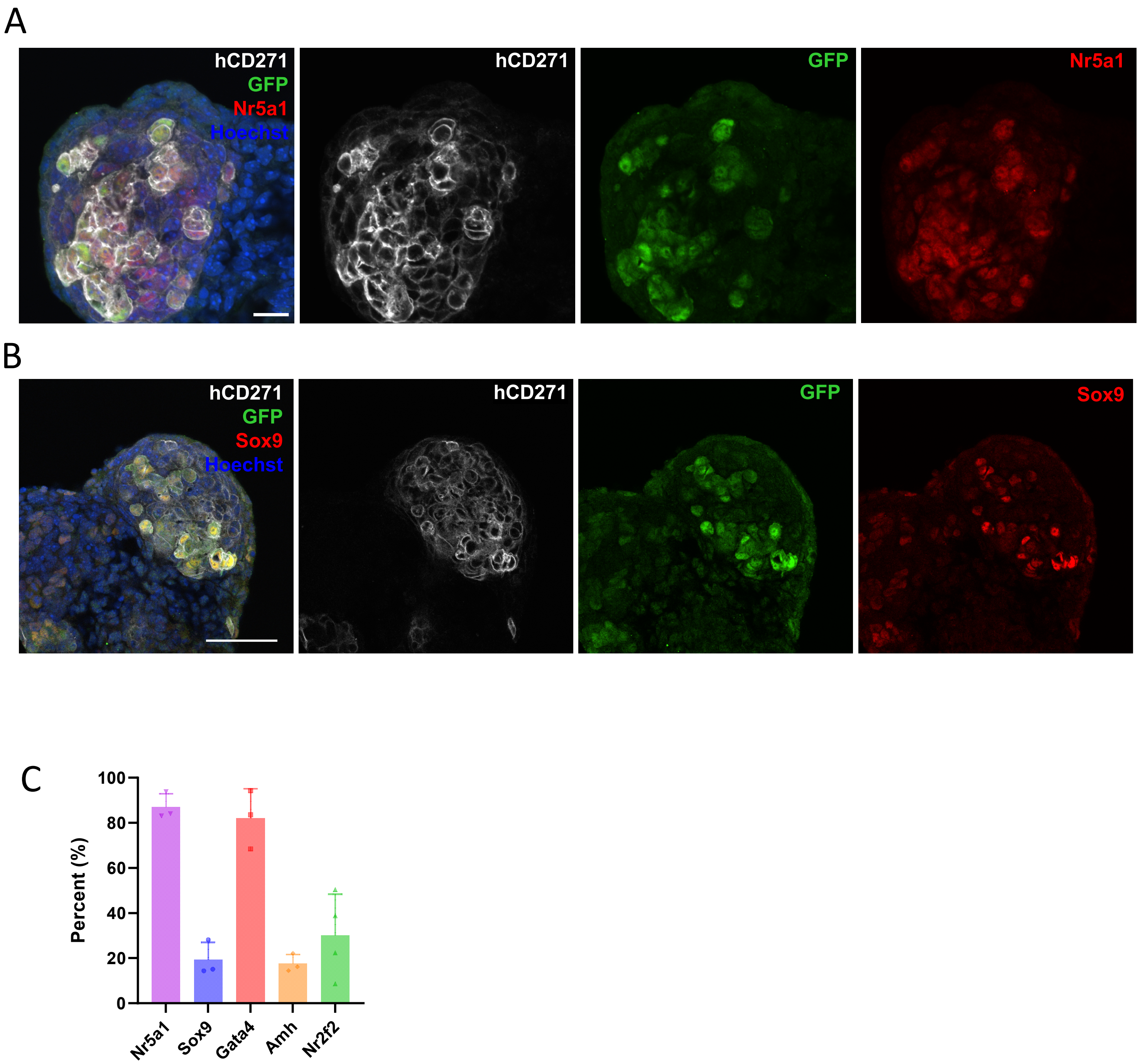

Fig. S4. Expression of marker genes in cell aggregates.
(A-B) Immunohistochemical staining of cryosections of D8 cell aggregates. Cryosections were labeled with anti-hCD271 antibody (white) and anti-GFP antibody (green), along with either anti-Nr5a1 antibody (red) (A) or Sox9 antibody (red) (B), and counterstained with Hoechst (blue). Scale bars = 50 µm. (C) The proportion of Nr5a1, Sox9, Gata4, Amh and Nr2f2-expressing cells among hCD271-positive cells was examined in cryosections.

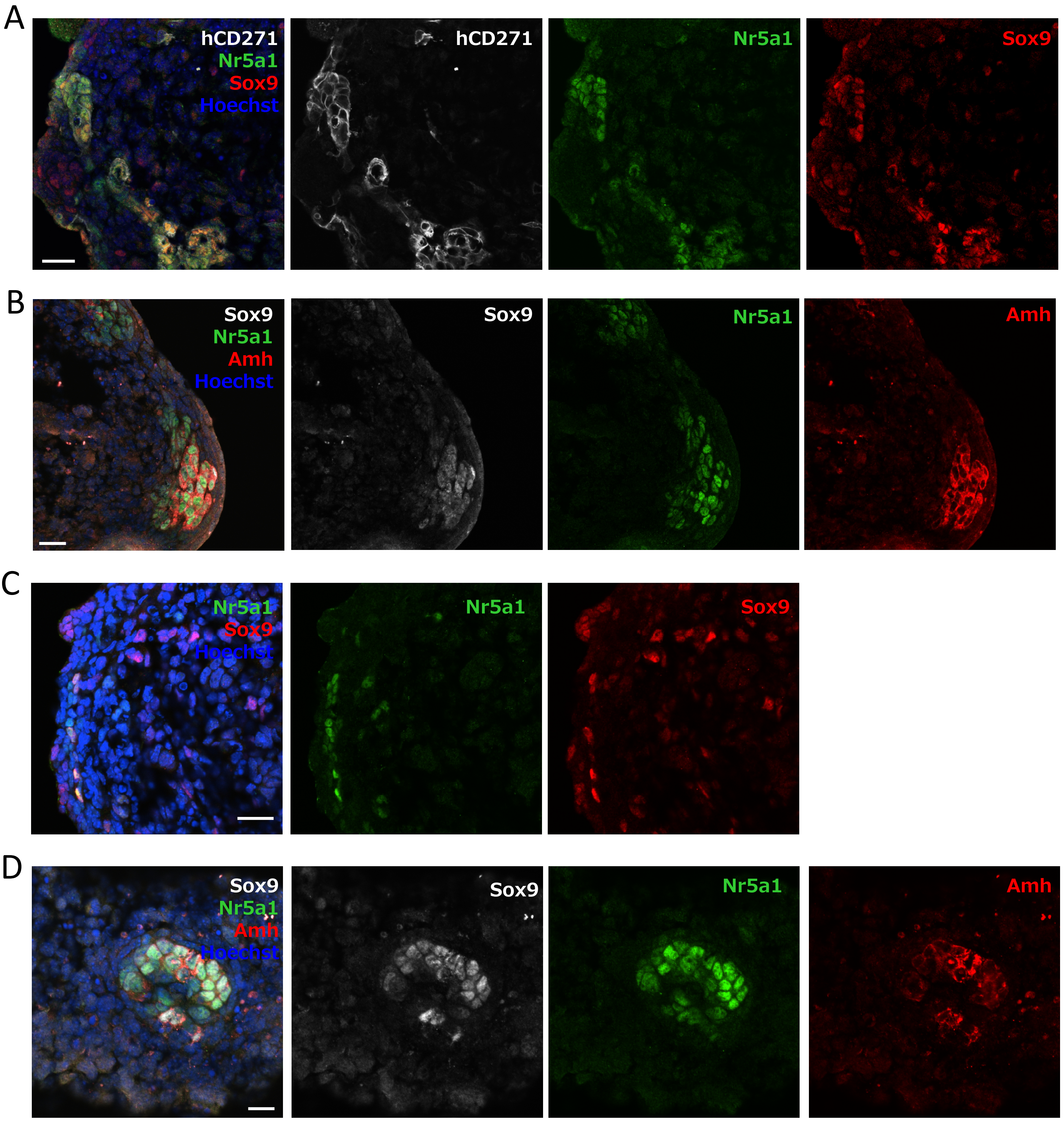

Figure S5. Differentiation of testicular somatic cells from additional ESC lines.
ESCs were subjected to the differentiation protocol to form cell aggregates, which were harvested at day 6 or day 8 and analyzed by immunostaining of cryosections. (A–B) Aggregates differentiated from a different clone of the Nr5a1-hCD271 ESC line were sectioned at day 8. (A) Sections were stained with anti-hCD271 (white), anti-Nr5a1 (green), and anti-Sox9 (red), with nuclei counterstained by Hoechst (blue). (B) Sections were stained with anti-Sox9 (white), anti-Nr5a1 (green), and anti-Amh (red), with nuclei counterstained by Hoechst. Expression of hCD271 was consistent with Nr5a1, and many cells also expressed Sox9. Furthermore, Amh expression was observed. (C–D) Aggregates differentiated from Amh-DTR ESCs, which have a different genetic background (mixture of ICR and C57BL/6), were analyzed at day 6 (C) and day 8 (D). (C) Nr5a1-positive cells and cells co-expressing Nr5a1 (green) and Sox9 (red) were detected. (D) Cells co-expressing Sox9 (white), Nr5a1 (green), and Amh (red), consistent with Sertoli-like cells, were observed. Nuclei were counterstained with Hoechst. Scale bars = 20 µm.

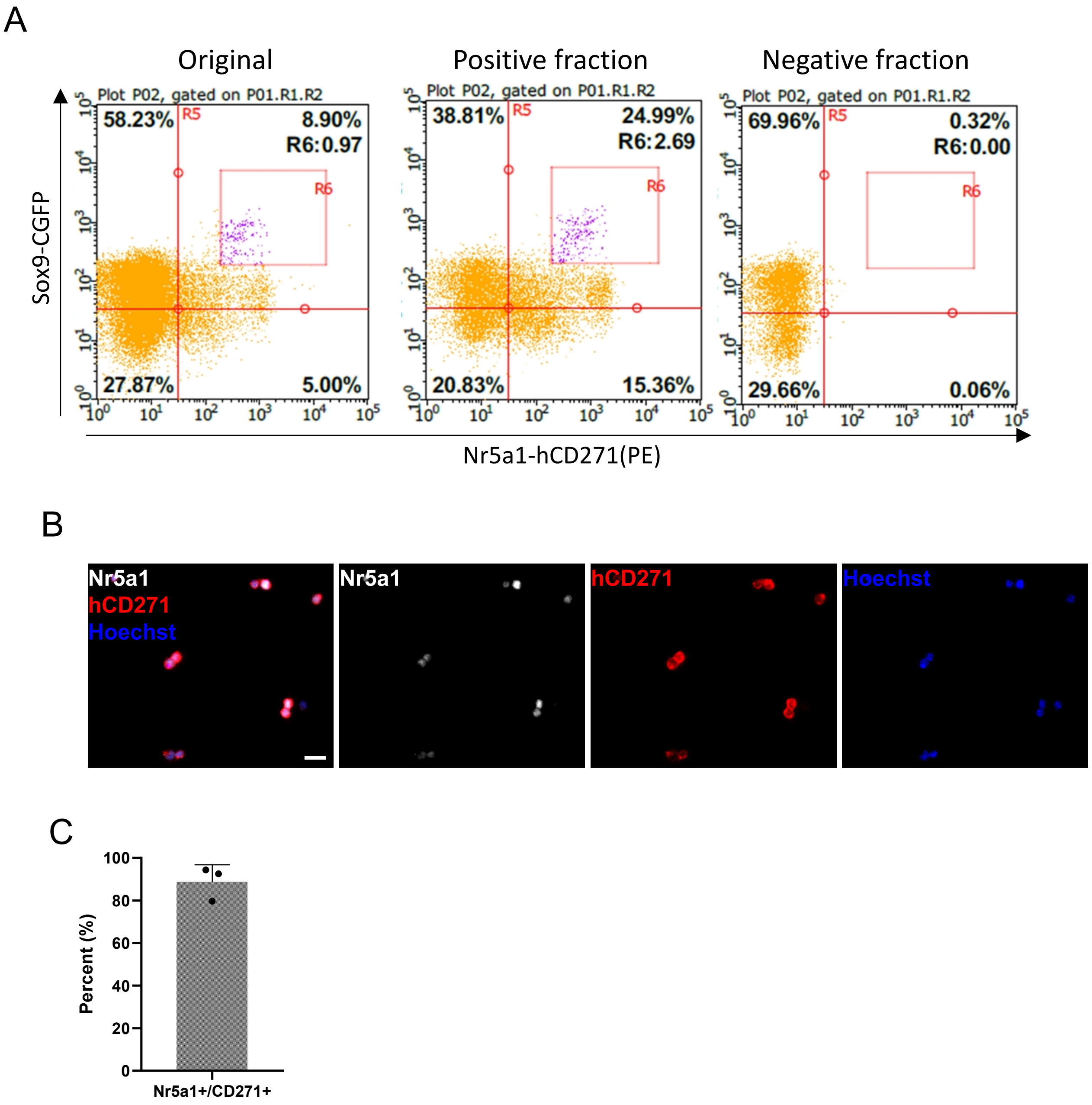

Figure S6. FCM and immunocytochemistry analysis of Nr5a1-hCD271-positive cells enriched by MACS.

(A) Cells induced from ESCs were subjected to magnetic-activated cell sorting (MACS) using an anti-hCD271 antibody at day 6 for subsequent scRNA-seq analysis. FCM plots are shown for the original unsorted cell population (left), the MACS-positive fraction (middle), and the MACS-negative fraction (right). The MACS-positive fraction was enriched for Nr5a1-hCD271-positive cells, although a subset of cells with low hCD271 expression remained. The MACS-positive fraction was used for scRNA-seq. (B) Immunocytochemistry of MACS-sorted cells. Cells were stained with antibodies against Nr5a1 (white) and hCD271 (red), and nuclei were counterstained with Hoechst (blue). Scale bar = 20 µm. (C) Quantification of Nr5a1-positive cells within the hCD271-positive cell population. A total of 300 cells were counted across three independent experiments. Most cells co-expressed hCD271 and Nr5a1, confirming the high concordance between reporter and endogenous Nr5a1 expression.

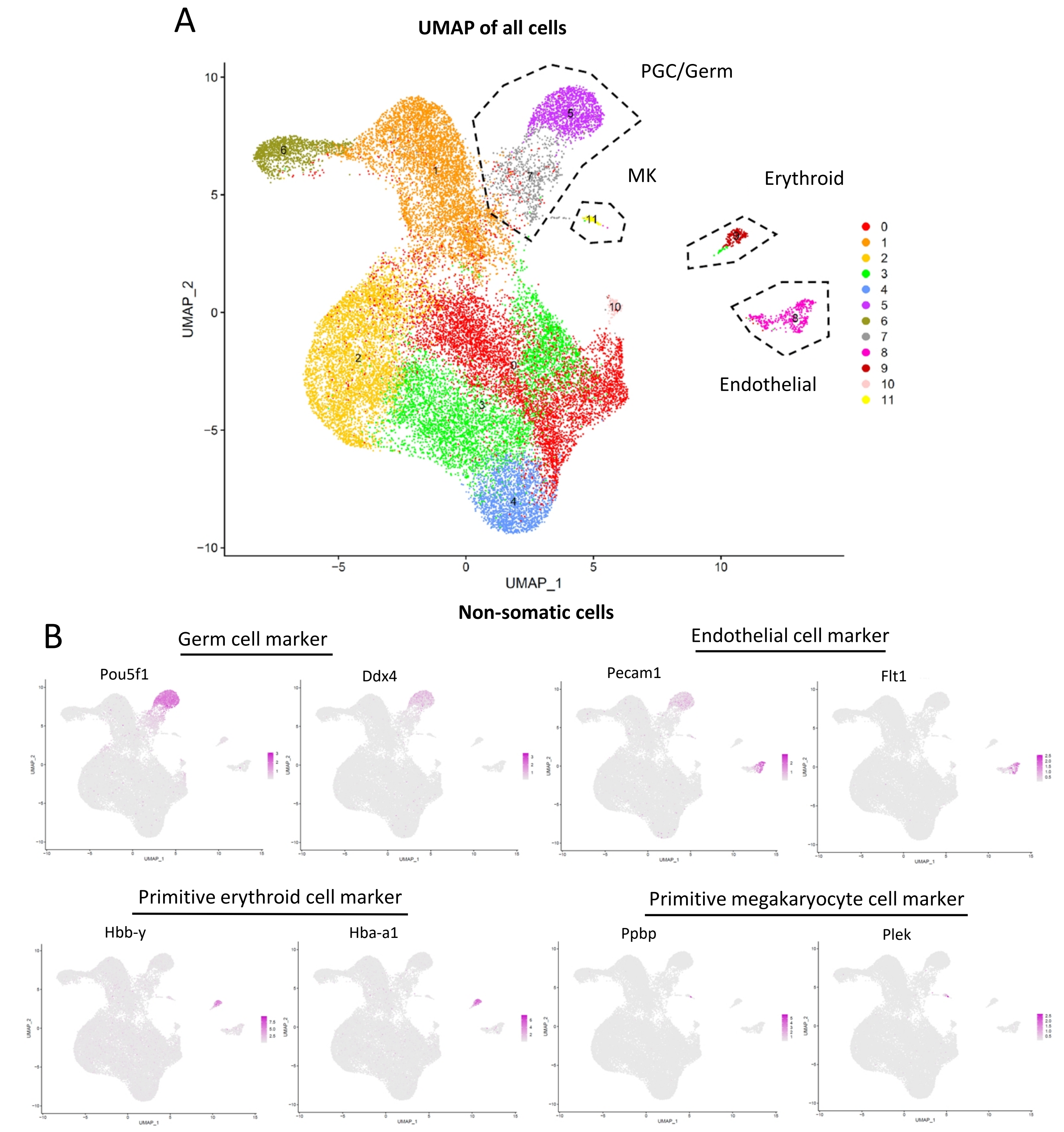

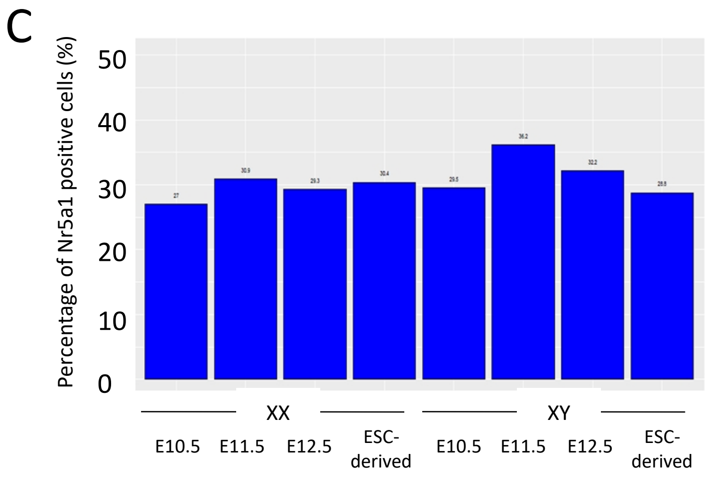

Figure S7. Comparative analysis of ESC–derived and in vivo gonadal cells

(A) UMAP plot generated from scRNA-seq data of in vivo gonadal cells (E10.5–E12.5) and in vitro ESC-derived cells. Male and female samples are combined. Clusters corresponding to germ cells, endothelial cells, primitive erythroid cells, and primitive megakaryocytes are outlined with dotted lines based on the data shown in (B). (B) Expression of marker genes for germ cells, endothelial cells, primitive erythroid cells, and primitive megakaryocytes, visualized using a magenta expression gradient. (C) Proportion of cells expressing endogenous *Nr5a1* within the gonadal somatic cell population.

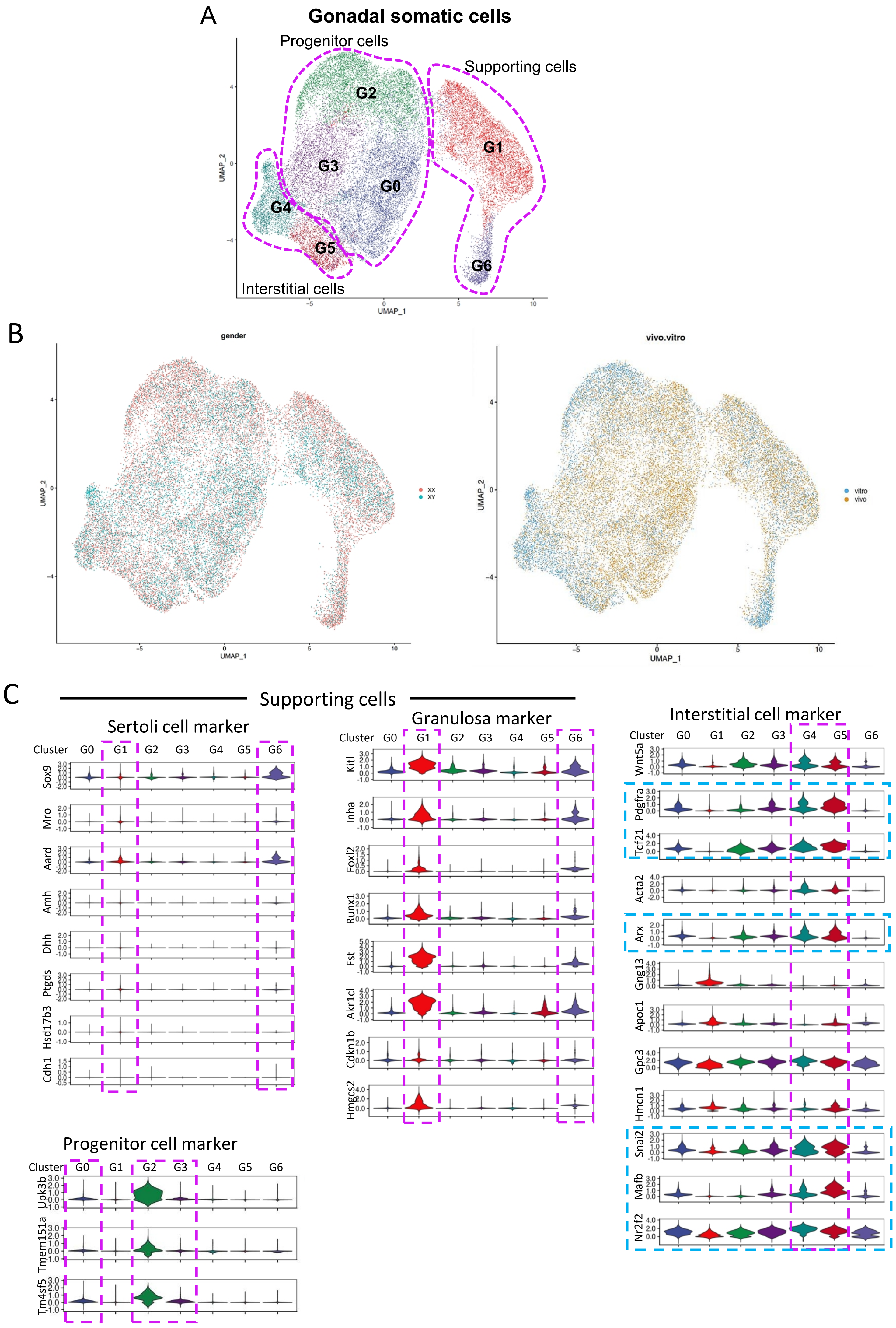

A
Gonadal somatic cells
Progenitor cells
Supporting cells
Interstitial cells
G0
G1
G2
G3
G4
G5
G6
UMAP_1
UMAP_2
B
gender
XX
XY
vivo.vitro
vitro
vivo
C
Supporting cells
Sertoli cell marker
Granulosa marker
Interstitial cell marker
Progenitor cell marker
Cluster
Sox9
Mro
Aard
Amh
Dhh
Ptgds
Hsd17b3
Cdh1
Kitl
Inha
Foxl2
Runx1
Fst
Akr1cl
Cdkn1b
Hmgcs2
Wnt5a
Pdgfra
Tcf21
Acta2
Arx
Gng13
Apoc1
Gpc3
Hmcn1
Snai2
Mafb
Nr2f2
Upk3b
Tmem151a
Tm4sf5

Fig. S8. Clustering of gonadal somatic cells.
(A) UMAP plot showing the classification of in vivo embryonic gonadal cells and ESC-derived in vitro gonadal cells. G1 and G6 were annotated as supporting cells (Sertoli or granulosa cells), G0, G2, and G3 as progenitor cells, and G4 and G5 as interstitial cells. (B) Violin plots showing the expression distributions of marker genes for supporting, interstitial, and progenitor gonadal somatic cells in each cluster. (C) UMAP representation colored by sex and sample (vivo vs vitro).

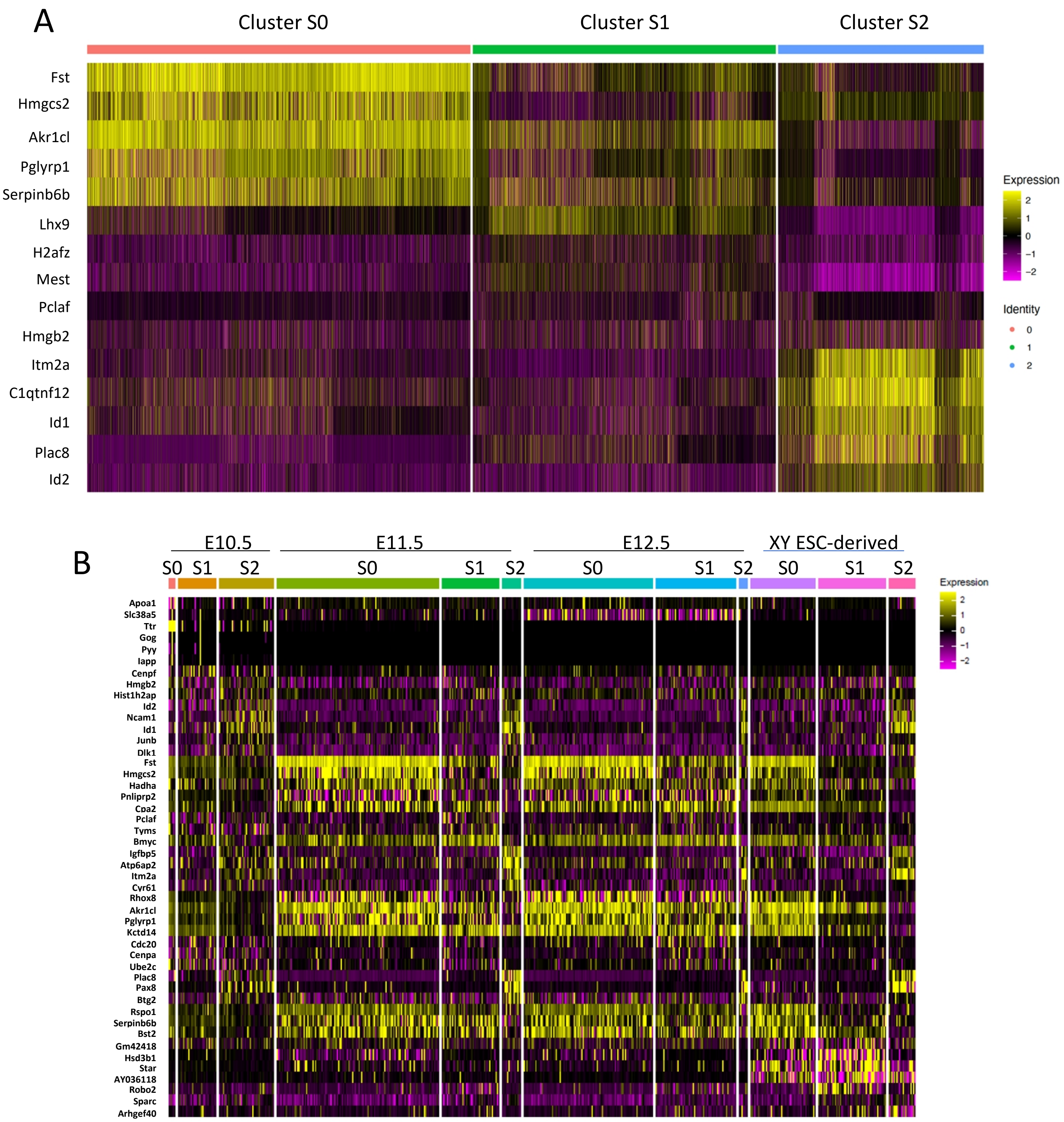

Fig. S9. Comparison of gene expression in supporting cell clusters.
(A) Heatmaps comparing gene expression profiles among clusters S0, S1, and S2 (in vivo and ESC-derived, male and female samples combined). The top five cluster-specific genes are shown. Expression levels are indicated by a magenta (low) to yellow (high) gradient, with black representing intermediate levels. (B) Heatmaps comparing gene expression profiles between in vivo testicular somatic cells (E10.5–E12.5) and XY ESC-derived cells, subdivided into clusters S0, S1, and S2. Compared to S0 and S2, the XY ESC-derived S1 cluster shows only partial resemblance to in vivo S1.

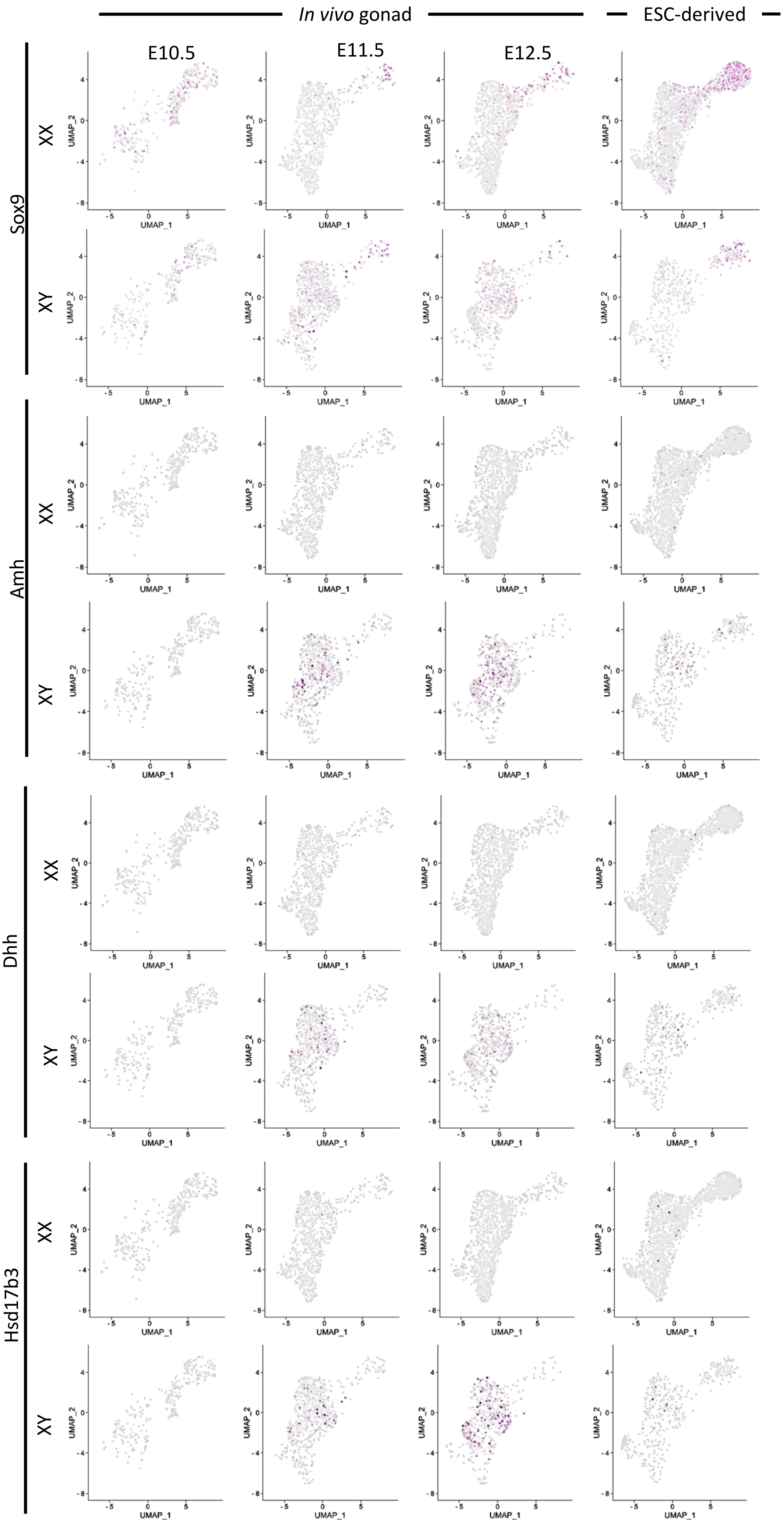
In vivo gonad
ESC-derived
E10.5
E11.5
E12.5
Sox9
Amh
Dhh
Hsd17b3
XX
XY
UMAP_1
UMAP_2

Fig. S10. Expression of male-specific genes in the UMAP plot of supporting cells

UMAP plot of supporting cells (adapted from Figure 3B), showing expression levels of *Sox9*, *Amh*, *Dhh*, and *Hsd17β3* using a magenta color gradient.

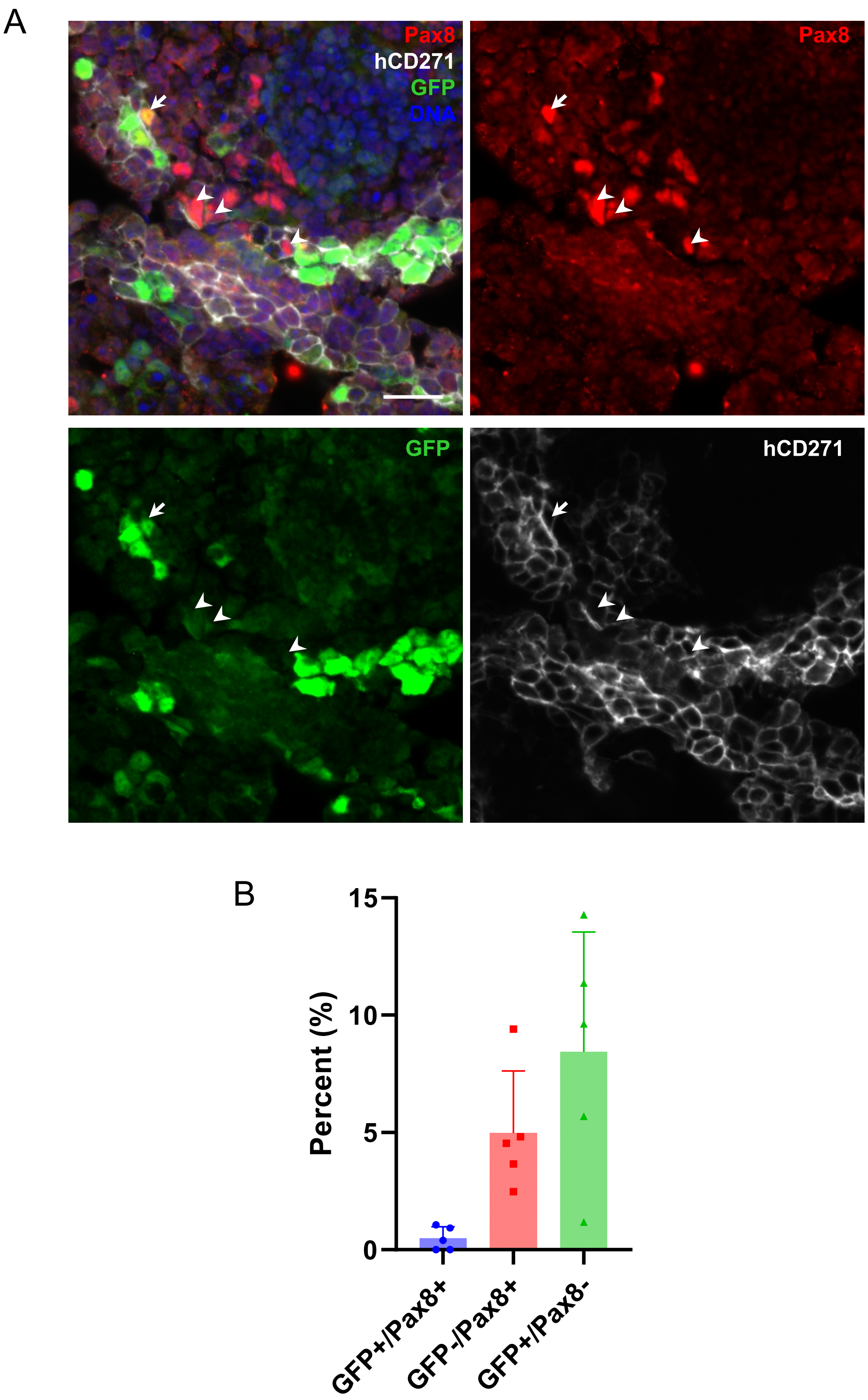

Fig.S11. Expression of Pax8 in D6 cell aggregate.

(A) Immunohistochemistry of D6 cell aggregates. Aggregates were stained with antibodies against Pax8 (red), hCD271 (white) and *Sox9*-GFP (green). Pax8+/GFP+/hCD271+ cells and Pax8+/GFP-(or weakly positive)/hCD271+ cells are indicated by arrows and arrowheads, respectively. Nuclei were counterstained with Hoechst (blue). Scale bar = 20 μm. (B) Quantification of the proportions of Pax8+/GFP+, Pax8+/GFP-, and Pax8-/GFP+ subpopulations among hCD271-positive cells. Data are presented as the mean ± SD (n=5 cell aggregates). The majority of Pax8-positive cells, putative supporting-like cells, exhibited negative or weak GFP expression. Conversely, most cells with high GFP expression were Pax8-negative and were therefore identified as Sertoli cells.

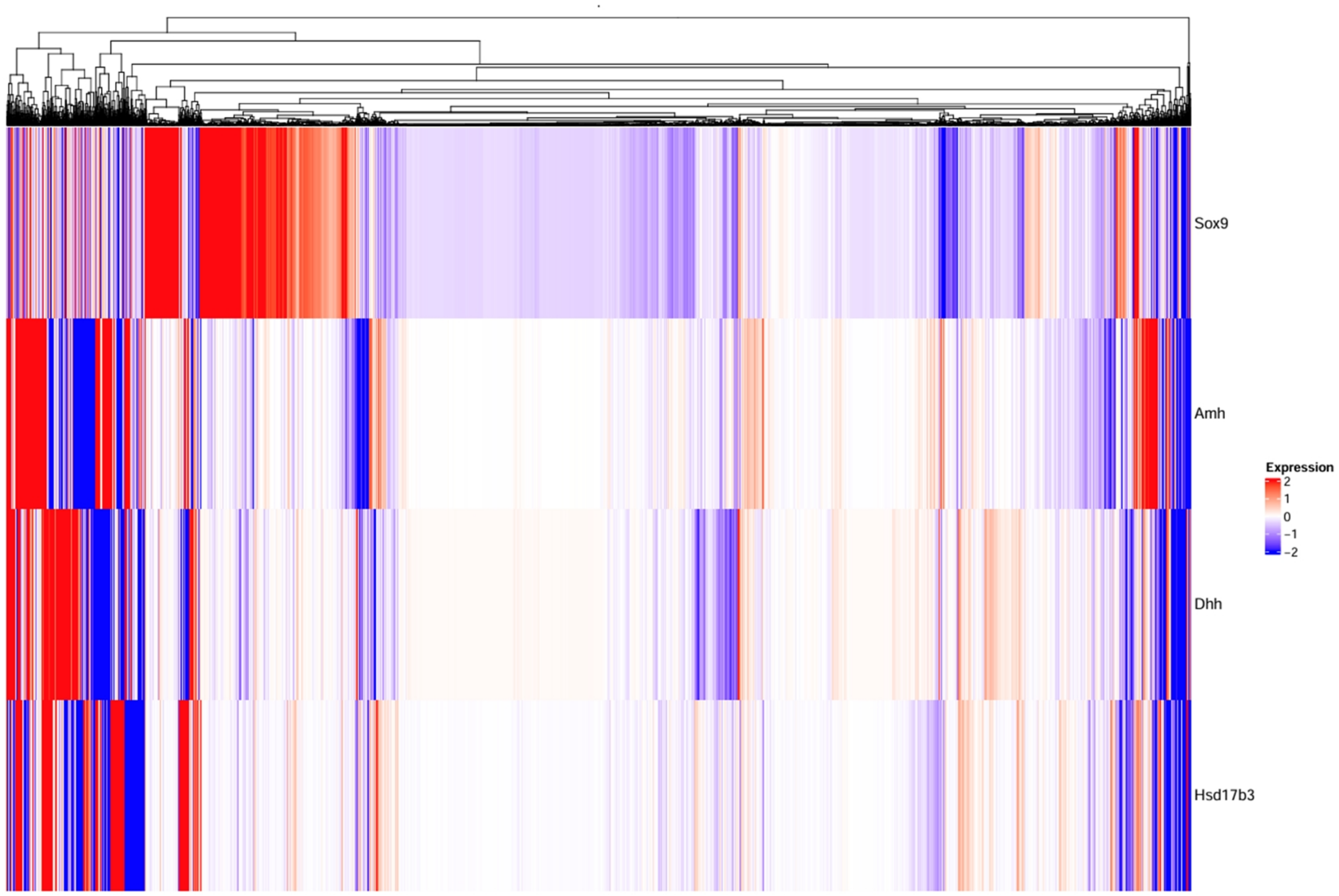

Fig. S12. Heatmap analysis of male-specific gene expression.
The heatmap visualizes the expression of the genes *Sox9*, *Amh*, *Dhh*, and *Hsd17β3* in the "Supporting cells" cluster of male ESC-derived cells. The cluster was predominantly composed of single- and double-positive cells, with very few triple- or quadruple-positive cells detected.

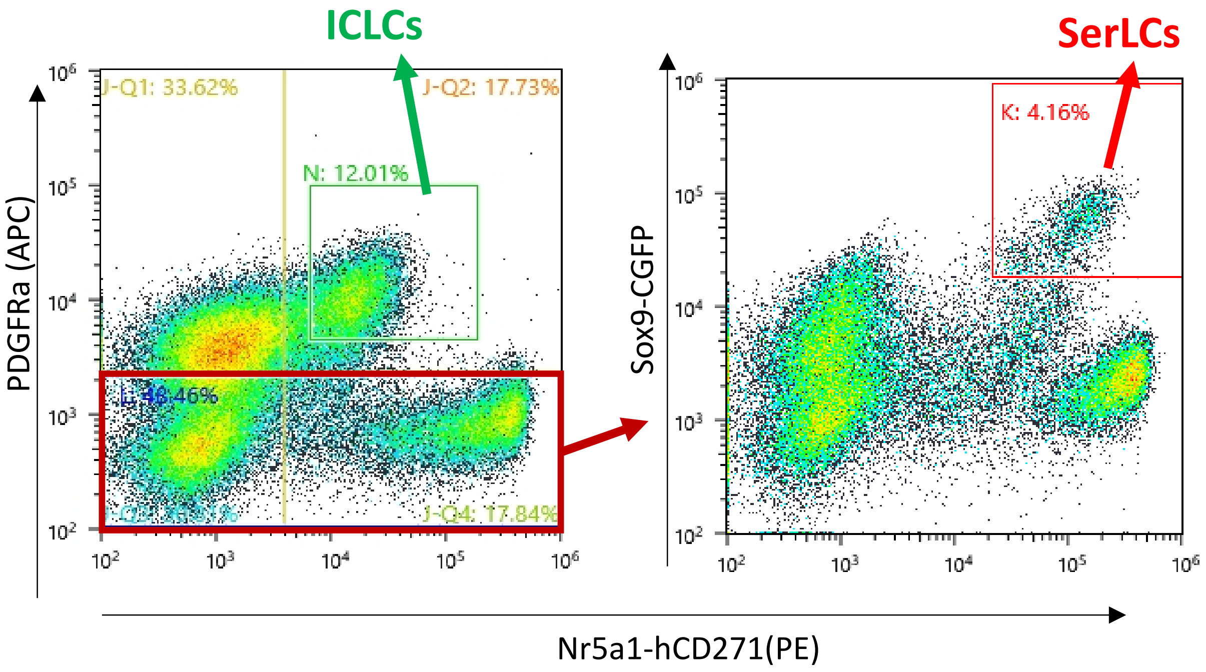

Figure S13. Sorting strategy for ICLCs and SerLCs

ICLCs were isolated as *Nr5a1*-hCD271^{low} / *PDGFRα*$^{+}$ cells by FACS. For SerLCs, cells were sorted as *Nr5a1*-hCD271^{high} / *Sox9*-CGFP^{high} cells using *PDGFRα*-negative gates.

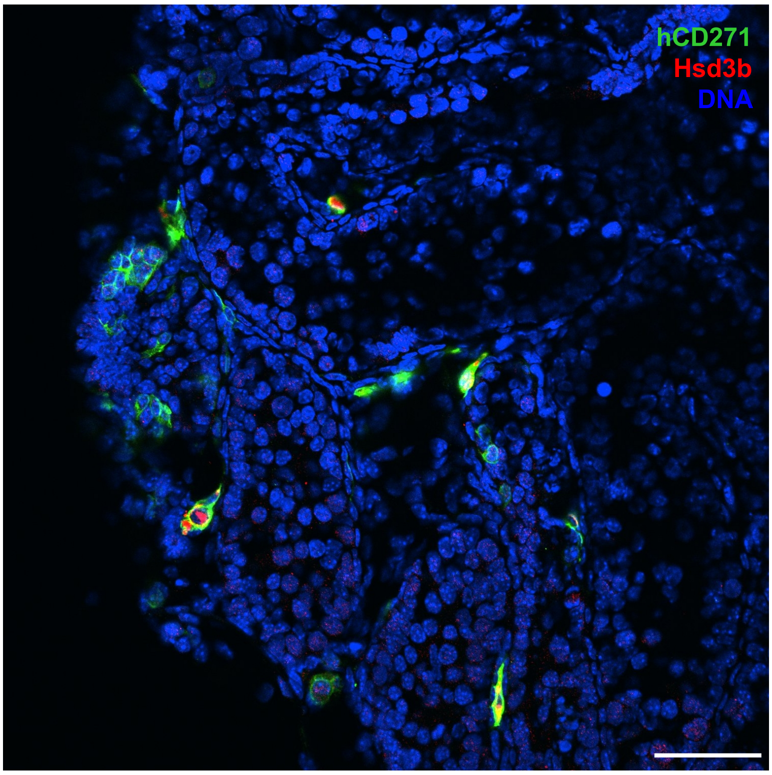

Fig.S14. Migration of ICLC into cultured *ΔFLE* testicular tissue.
Immunofluorescence images of cryosections from *ΔFLE* testicular tissues supplemented with ICLCs after 7 days of culture. Sections were stained with antibodies against hCD271 and HSD3b, and nuclei were counterstained with Hoechst. ICLCs were identified as hCD271-positive cells located in the peripheral and interstitial regions of the tissue. Scale bar, 50 μm.

A

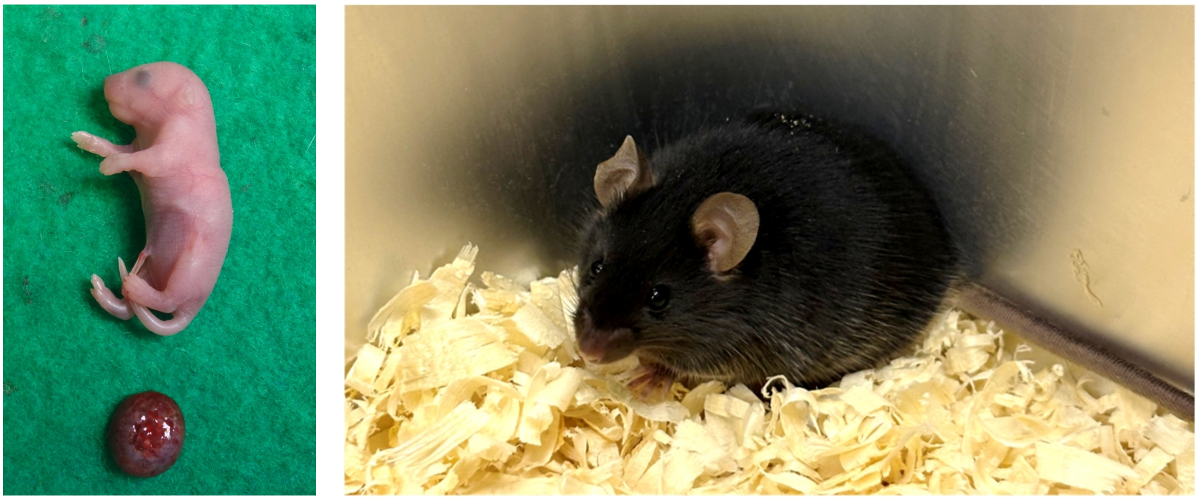

B

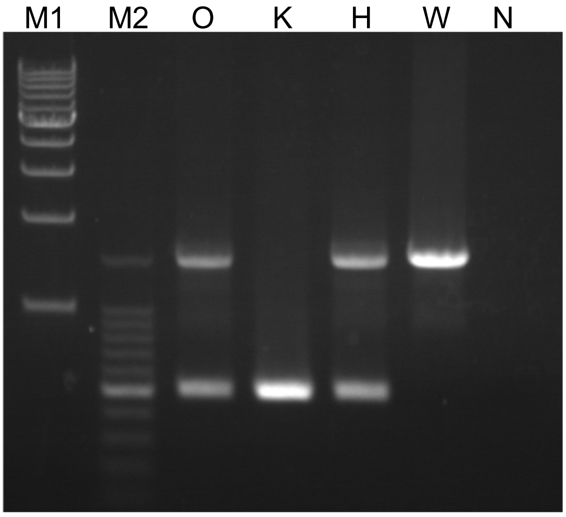

Fig. S15. Offspring from ΔFLE mice

(A) A single male offspring obtained from ROSI performed on spermatid cells obtained from ICLC-supplemented ΔFLE mouse testes. The photo on the left shows the pup immediately after birth, and the photo on the right shows it after weaning. It grew up healthy. (B) Genotyping results of offspring. The wild-type and ΔFLE alleles are detected as 1493 bp and 615 bp bands, respectively. Lanes show a 1 kbp ladder marker (M1), 100 bp ladder marker (M2), offspring (O), positive control (ΔFLE KO (K), ΔFLE hetero (H) and wild type (W)), and Non-template control (N). The offspring were heterozygous, which was the expected outcome given that spermatids from ΔFLE mice were used to fertilize wild-type oocytes.

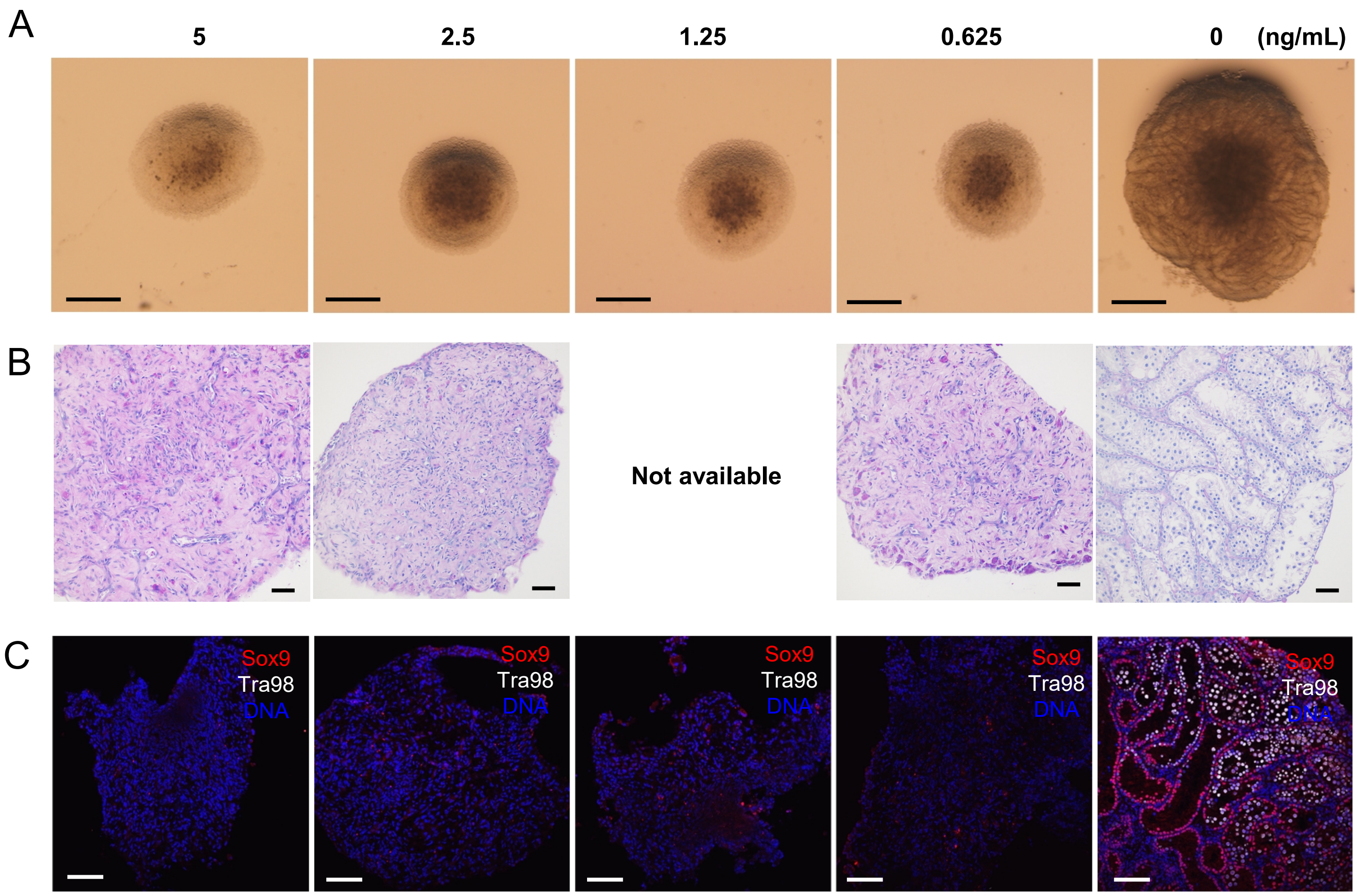

Fig. S16. Stepwise re-evaluation of DT concentration for Sertoli cell ablation from neonatal Amh-DTR mouse testes.

(A) Stereomicroscopic images of testicular tissues from a 4.5 dpp mouse cultured for 19 days. Tissues were cultured for the first week in medium containing DT at concentrations of 5, 2.5, 1.25, 0.625, or 0 ng/mL, respectively, and subsequently maintained in regular, DT-free medium. (B) Periodic acid–Schiff (PAS) staining of the testicular tissues shown above. Scale bars = 50 µm. (C) Immunostaining of the testicular tissues shown above. Sections were labeled with antibodies against Sox9 (Sertoli cells) and Tra98 (germ cells) and counterstained with Hoechst (nuclei). Scale bars = 100 µm. The central panel is marked as “Not available” due to a technical issue during sectioning. To stepwise evaluate the efficiency of Sertoli cell ablation, a serial dilution of DT was prepared in the culture medium, ranging from 5 ng/mL to 0.625 ng/mL. The results confirmed that Sertoli cells were successfully ablated even at a concentration one-eighth of the 5 ng/mL dose typically used in the in vitro Sertoli cell replacement method.

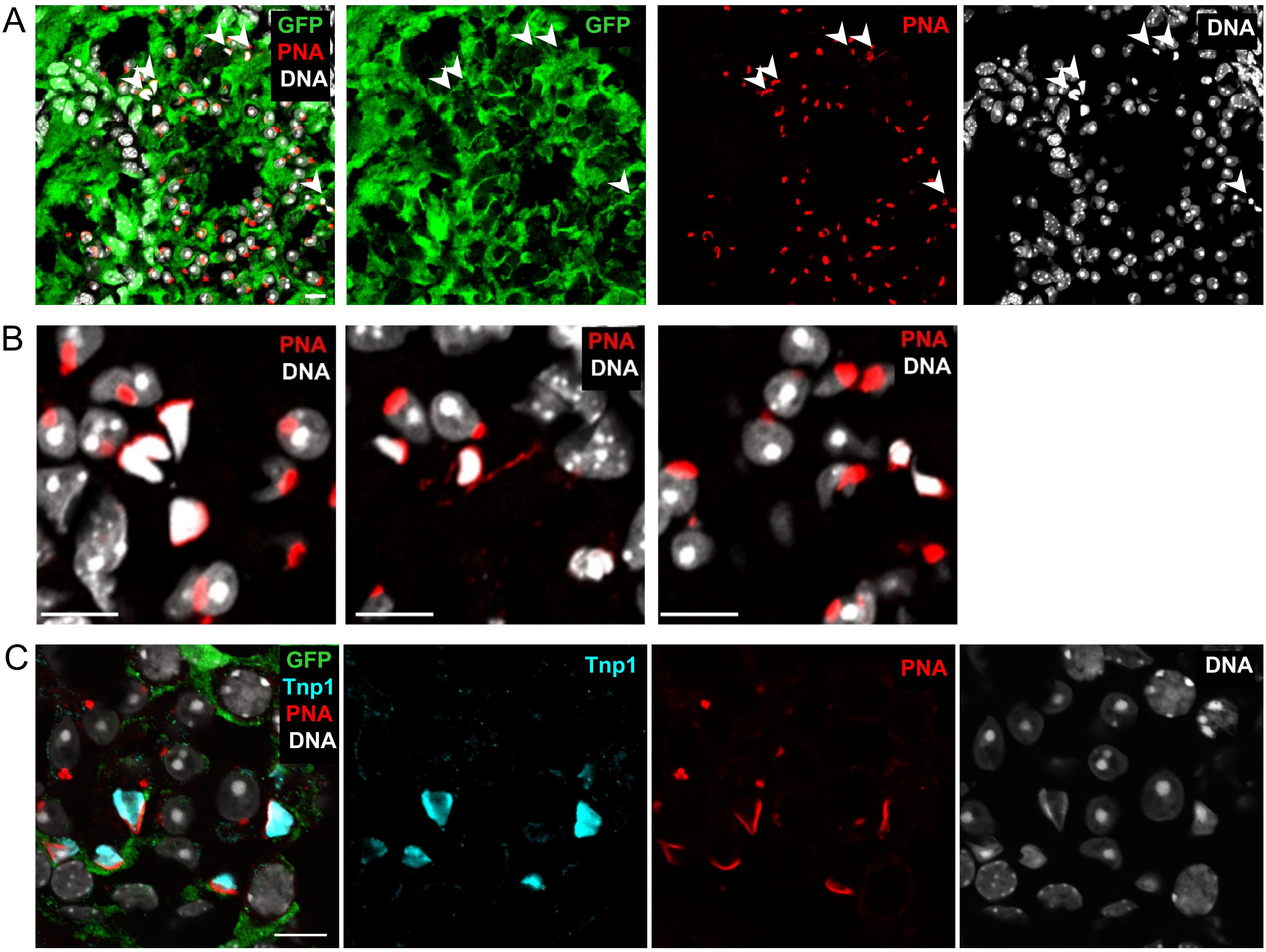

Fig. S17. Progression of spermatogenesis in reconstituted seminiferous epithelium. (A) Immunostaining of reconstituted seminiferous epithelium after 35 days of culture, with images of the merged and individual channels shown. The tissue was labeled with an anti-GFP antibody (green) and PNA (red), with nuclei counterstained by Hoechst (white). Within the GFP-positive tubules (indicating their SerLC origin), round spermatids and elongating spermatids (arrowhead) were observed. Scale bar = 10 μm. (B) A merged image of the PNA and DNA (Hoechst) channels, showing a magnified view of the cell indicated by the arrowhead in (A). Scale bar = 10 μm. (C) Immunostaining for Transition protein-1 (Tnp1) to identify late-stage spermatids. The image shows reconstituted seminiferous epithelium after 35 days of culture, with merged and individual channels. The tissue was labeled with an anti-GFP antibody (green), and with an anti-Tnp1 antibody and PNA (red). Nuclei were counterstained with Hoechst (white). Tnp1 is a gene expressed at steps 12-13 of spermatid development. Scale bar = 10 μm.

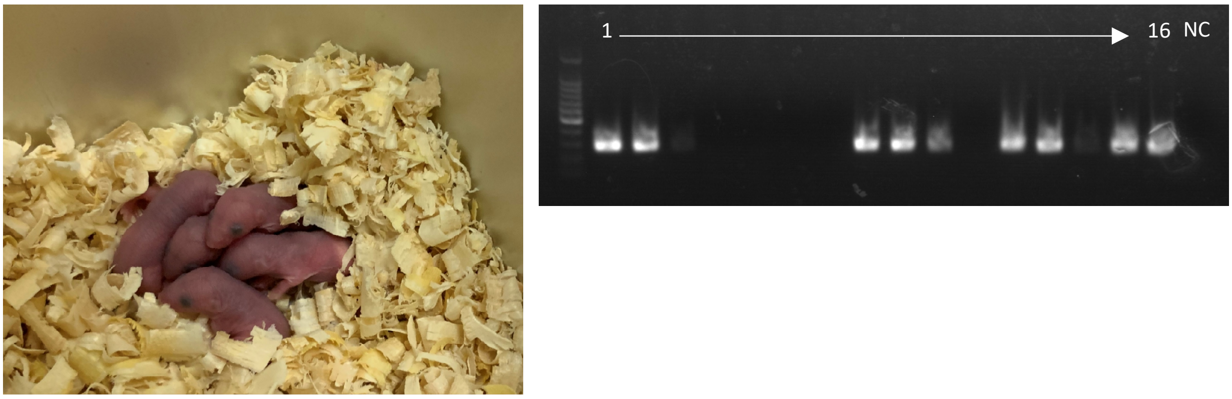

Figure S18. Fertility of offspring obtained by ROSI

F2-generation offspring obtained by natural mating of the female mouse obtained by ROSI with a wild-type male. A total of sixteen offspring were born across two litters. The image on the right shows PCR genotyping of the F2 offspring using primers specific to the *Amh*-DTR transgene, which was detected as a 282 bp band.

Table S1. Summary of microinsemination experiments for ΔFLE mouse

Sample ID	ICSI/ROSI	Number of oocytes inseminated	Number of oocytes developing into 2-cell embryos	Number of live offspring
0430 ILC3-1	ROSI	130	77	1

Table S2. SerLC-transplanted testicular tissue showing the presence of spermatocytes and spermatids upon immunostaining of cryosections.

Experiment ID	Tissue ID	Spermatocytes	Spermatids
igst1014	5-3	+	+
igst1209	2	-	-
igst0113	1-1	+	+
	3-1	+	+
	3-2	+	+
igst0121	1-1	+	+
igst0204	3-1	+	+
	3-2	+	+
	3-3	+	+
igst0527	1-1	-	-
	1-4	+	+
igst0603	1-1	+	+
	2-4	+	+
	3-1	+	+

Table S3. Summary of microinsemination experiments

Sample ID	ICSI/ROSI	Number of oocytes inseminated	Number of oocytes developing into 2-cell embryos	Number of live offspring
0113 1-5	ROSI	60	42	0
0113 5-1	ROSI	8	4	0
0113 3-3	ROSI	77	68	1

*All of embryos were transferred into six pseudopregnant ICR females.

Table S4. Summary of fertility test of F1 mouse

Date of birth	Number of offspring	Male/Female	Number of Tg mouse
2022/7/22	6	3/3	2
2022/8/16	10	4/6	7

Table S5. Oligonucleotides used in this study

Target	Purpose	Sequence	Band size
Zfy	Genotyping (sex determination)	CCTATTGCATGGACTGCAGCTTATG<br>GACTAGACATGTCTTAACATCTGTCC	184bp
Sox17	Genotyping (sex determination , positive control)	CCCTTAAGGCCGTAGTACAGGTGCAGAGC<br>GCCGCGTGGCCATGGATGGC	382bp
b-actin	qPCR	GATCTGGCACCACACCTTCT<br>GGGGTGTTGAAGGTCTCAAA	138bp
Amh	qPCR	CTATTTGGTGCTAACCGTGGACTT<br>AAGGCTTGCAGCTGATCGAT	204bp
Foxl2	qPCR	CACCTTGATGAAGCACTCGT<br>ATCATAGCCAAGTTCCCGTTC	96bp
Dhh	qPCR	ACCCCGACATAATCTTCAAGGAT<br>GTACTCCGGGCCACATGTTC	120bp
HSD17b 3	qPCR	ATGGAGTCAAGGAGGAAAGGC<br>GGCTGTAAAGAGGCCAGGG	76bp
Fst	qPCR	CCAGGCAGCTCCACTTGTGT<br>AGTCACTCCATCATTTCCACAAAG	120bp
Rspo1	qPCR	CTCCGACACCAAAGAGACCC<br>GTTGCCCTTTGTGTCTCCGA	167bp
Sox9	guide RNA	CCCTGAGAAGAGAAAAGCTA	
Sox9-CGFP	Genotyping	TCCCCGCAACAGATCTCCTA<br>AGCTCACCAATGCTCTATGTT<br>AGCAGGCTGGAGACGTGGAGGAGAACCCT<br>GGACCTATGGTGAGCAAGGGCGAG	Wild type: 1202 bp<br>Knock-in: 1950 bp
Nr5a1-hCD271	Genotyping	CACAGACCAGGGCAATCCCAAGCC<br>GTCGGAGAACGTCACGCTGTCCAG	300 bp
Amh-DTR	Genotyping	AGAAAGGGCTCTTTGAGAAGGCCACTCTGC<br>CCATTCTAAACAACACCCTGAAAACTTTGC	282 bp
ΔFLE (Shima et al., 2018 Development) *	Genotyping	TTAGAGCATGCAGGTATAGCAGAG<br>AAGGGATGAACACTAAGGGGTT<br>CTGTTGAATGCATATCTGAAGGTC	Wild type: 1493 bpΔFLE: 615 bp

*We partially modified the primer set from the previous study. For the wild-type allele, this primer set primarily yields a 1493 bp band. A larger, 2899 bp band is also possible but is rarely detected due to inefficient amplification.
